# Effects of methylphenidate on reinforcement learning depend on working memory capacity

**DOI:** 10.1007/s00213-021-05974-w

**Published:** 2021-10-21

**Authors:** Mojtaba Rostami Kandroodi, Jennifer L. Cook, Jennifer C. Swart, Monja I. Froböse, Dirk E. M. Geurts, Abdol-Hossein Vahabie, Majid Nili Ahmadabadi, Roshan Cools, Hanneke E. M. den Ouden

**Affiliations:** 1 School of Electrical and Computer Engineering, College of Engineering, University of Tehran, Tehran, Iran; 2 Donders Institute for Brain, Cognition and Behaviour, Radboud University, Nijmegen, The Netherlands; 3 School of Psychology, University of Birmingham, Birmingham, United Kingdom; 4 Department of Psychiatry, Radboud University Medical Centre, Nijmegen, The Netherlands; 5 School of Cognitive Sciences, Institute for Research in Fundamental Sciences (IPM), Tehran, Iran

**Keywords:** Reversal learning, Methylphenidate, Catecholamines, Dopamine, Working memory, Computational modelling of behaviour

## Abstract

**Rationale:**

Brain catecholamines have long been implicated in reinforcement learning, exemplified by catecholamine drug and genetic effects on probabilistic reversal learning. However, the mechanisms underlying such effects are unclear.

**Objectives and methods:**

Here we investigated effects of an acute catecholamine challenge with methylphenidate (20 mg, oral) on a novel probabilistic reversal learning paradigm in a within-subject, double-blind randomised design. The paradigm was designed to disentangle effects on punishment avoidance from effects on reward perseveration. Given the known large individual variability in methylphenidate’s effects, we stratified our effects by working memory capacity and trait impulsivity, putatively modulating the effects of methylphenidate, in a large sample (*n* = 102) of healthy volunteers.

**Results:**

Contrary to our prediction, methylphenidate did not alter performance in the reversal phase of the task. Our key finding is that methylphenidate altered learning of choice-outcome contingencies in a manner that depended on individual variability in working memory span. Specifically, methylphenidate *improved* performance by adaptively *reducing* the effective learning rate in participants with higher working memory capacity.

**Conclusions:**

This finding emphasises the important role of working memory in reinforcement learning, as reported in influential recent computational modelling and behavioural work, and highlights the dependence of this interplay on catecholaminergic function.

**Supplementary Information:**

The online version contains supplementary material available at 10.1007/s00213-021-05974-w.

## Introduction

Brain catecholamines (dopamine and noradrenaline) are well known to play a fundamental role in reinforcement learning and decision-making. Most notably, in the last 2 decades, a wealth of studies have shown that dopaminergic midbrain firing increases when experience exceeds expectations (Montague et al. 1996; Fiorillo et al. 2003; Schultz 2016). This dopaminergic signalling is widely accepted to function as a teaching signal driving reinforcement learning (Niv and Montague 2009). Dopamine has also been implicated in the ability to flexibly adjust behaviour to changing environments (Swainson et al. 2000; Cools et al. 2001; Chudasama and Robbins 2006; Dodds et al. 2008; Boulougouris et al. 2009; Clatworthy et al. 2009; Clarke et al. 2011; Cools and D’Esposito 2011; Groman et al. 2012; den Ouden et al. 2013). For example, selective lesioning of striatal dopamine in marmoset monkeys impaired the ability to reverse learnt stimulus-reward associations (Clarke et al. 2011), in line with classic findings from studies in rodents showing that reversal learning is altered by dopaminergic modulation of the (ventral) striatum (Taghzouti et al. 1985; Smith et al. 1999; Goto and Grace 2005). In patients with Parkinson’s disease, dopaminergic medication has been shown to impair performance selectively on the reversal learning stage of a probabilistic reversal learning task while leaving learning during an initial acquisition phase unaltered (Cools et al. 2001). In line with the proposal that this impairment reflects detrimental overdosing of relatively intact dopamine levels in the ventral striatum, dopaminergic medication in Parkinson’s disease was shown to attenuate reversal-related BOLD signal in the ventral (but not dorsal) striatum (Cools et al. 2007a). Subsequent studies in young healthy volunteers have shown that administration of the dopamine (and noradrenaline) transporter blocker methylphenidate to healthy volunteers modulates reversal-related BOLD signal in the striatum (Dodds et al. 2008) and impaired reversal learning in proportion to the degree that methylphenidate increased striatal dopamine release (Clatworthy et al. 2009). While these studies establish a causal role for striatal dopamine specifically in reversal learning, the mechanism by which dopamine alters the ability to reverse responding remains unclear.

In the present study, we aimed to elucidate the nature of the catecholaminergic effects on perseverative behaviour during reversal learning. Specifically, perseveration may result from either a ‘stamping in’ of rewarded behaviour, leading to an inability to ‘let go’ of responding to a previously rewarded stimulus, or from a changed ability to approach a previously punished stimulus (i.e. punishment avoidance). While striatal Go/NoGo pathway models, including the Opponent Actor Learning model, posit that perseveration might follow from both increasing the impact of reward and reducing the impact of punishment (Frank 2005; Collins and Frank 2014), we hypothesised that reversal deficits are more likely to follow from disproportionate stamping in of rewarded behaviour, rather than from diminished punishment avoidance. This hypothesis is grounded in seminal work with experimental rodents, showing that injection of D-amphetamine in the nucleus accumbens of rats potentiates behavioural control by stimuli formerly associated with reward (i.e. conditioned reinforcement) in a DA-dependent way (Robbins et al. 1989; Parkinson et al. 1999). Moreover it follows directly from our prior genetic study of probabilistic reversal learning (den Ouden et al. 2013). Specifically, we have shown that perseveration elicited by genetic variability in the dopamine transporter *DAT1* can be accounted for by progressively increased reliance on prior reinforcement, captured by an increase in an experience-weight parameter in an augmented reinforcement learning model (den Ouden et al. 2013). Thus, natural genetic *DAT1* variation was associated with a stronger correlation between reinforcement history and perseveration (den Ouden et al. 2013). Accordingly, we hypothesised that increasing catecholamine signalling would alter perseverative behaviour, specifically by inducing an inability to ‘let go’ (i.e. stop choosing) a previously rewarded stimulus rather than by impairing the ability to approach a previously punished stimulus (Frank et al. 2004; Cools et al. 2006).

To dissociate these two alternative mechanisms of perseverative behaviour arising from punishment avoidance or excessive adherence to previously rewarded stimuli, we introduce a novel reversal learning paradigm that included a ‘neutral’ choice option. To assess whether and which of these mechanisms are affected by catecholamine signalling, we combined this novel paradigm with administration of catecholamine transporter blocker methylphenidate, which acts by blocking dopamine and noradrenaline transporters (DAT/NAT). This blockade increases extracellular catecholamine availability in the synaptic cleft, without stimulating release or acting as a receptor (ant)agonist (Volkow et al. 2002). It is thought to prolong the effect of both dopamine and noradrenaline release, as reuptake is slowed (Madras et al. 2005; Berridge et al. 2006).

Finally, previous studies have shown that there is large inter-task and inter-individual variability in catecholaminergic drug effects on cognitive task performance (Kimberg et al. 1997; Cools et al. 2007b; Van Der Schaaf et al. 2013; Linssen et al. 2014; Swart et al. 2017; Froböse et al. 2018; Cook et al. 2019), including probabilistic reversal learning (Clatworthy et al. 2009). Given that methylphenidate prolongs the effects of catecholamine release by blocking the reuptake of catecholamines, it is likely that the effect of methylphenidate on catecholamine-dependent function is a function of dopamine synthesis capacity and release. Simply put, if there is no release, there is no reuptake to block. To take into account the established large individual variability in methylphenidate effects, we collected a large sample (*n* = 102) to expose individual differences and stratified methylphenidate effects by two measures that have been previously demonstrated to relate to baseline dopamine function: working memory (WM) span for its relation to striatal dopamine synthesis capacity (Cools et al. 2008; Landau et al. 2009) and trait impulsivity for its relation to dopamine (auto)receptor availability (Lee et al. 2009; Buckholtz et al. 2010; Reeves et al. 2012; Kim et al. 2014). Based on previous studies where we used methylphenidate in combination with various reinforcement learning tasks (Van Der Schaaf et al. 2013; Swart et al. 2017; Cook et al. 2019), we hypothesised that WM span specifically would predict the inter-individual differences in reversal learning.

## Methods

### General procedure and pharmacological intervention

Data was collected April 15–September 1, 2014, and took place at the Donders Institute for Brain, Cognition and Behaviour, Centre for Cognitive Neuroimaging. The study consisted of two test sessions with an interval of 1 week to 2 months. The first test day started with informed consent, followed by a medical screening. Participation was discontinued if participants met any of the exclusion criteria (supplemental methods 3). On both test days, participants first completed baseline measures, as well as the Instrumental and Pavlovian phases of the Pavlovian-Instrumental transfer (PIT) task (Geurts et al. 2021). Participants received a capsule containing either 20 mg of catecholamine transporter blocker methylphenidate (Ritalin®, Novartis) or placebo, in a double-blind, placebo-controlled, cross-over design. This relatively low dose was selected because (i) this minimises any potential risks, (ii) it has been found sufficient to affect cognitive performance and to do so in a manner that is indistinguishable from administration of a higher (40 mg) dose (e.g. Elliott et al. 1997), and (iii) dopamine microdialysis in macaque monkeys has shown that low dose of MPH leads to relatively preferential effects on striatal (relative to prefrontal) DA release (Kodama et al. 2017). When administered orally, methylphenidate has a maximal plasma concentration after 2 h and a plasma half-life of 2–3 h (Kimko et al. 1999). Below we denote capsule intake as *t* = 0.

**Fig. 1:**
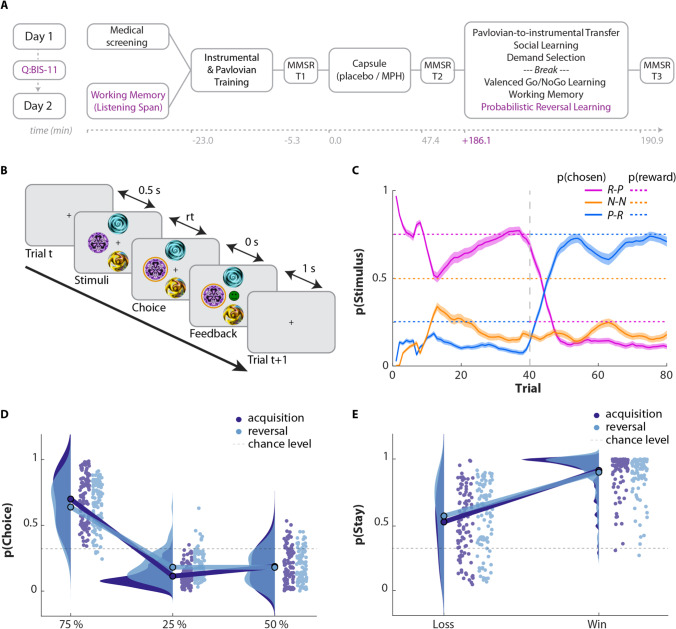
Experimental design and basic results. **A** Study timeline. Participants took part in 2 sessions, where on day 1, they started with a 20-min medical screening and on day 2 completed a working memory test (listening span). Neuropsychological questionnaires (Quest) were completed at home between the two sessions. Mood and medical symptom ratings (MMSR) were acquired at 3 time points. A battery of 6 tasks (Swart et al. 2017; Froböse et al. 2018; Cook et al. 2019) was performed in a fixed order, where the probabilistic reversal learning (PRL) task was always performed last. Average timings are indicated, with timings most relevant for the current study in purple. **B** Reversal learning design. On each trial, three visual stimuli were represented in three out of four randomly selected locations. Participants had to choose one of the stimuli with a mouse click and subsequently received feedback. The feedback would either be a reward (green, happy emotion) and punishment (red, sad emotion). During acquisition, the rewarded stimulus (here purple) resulted in a 75:25 ratio of reward/punishment. Selecting the neutral stimulus (orange) and punished stimulus (blue) led to 50:50 and 25:75 ratio of reward/punishment, respectively. After 40 trials, the reversal phase started, and rewarded and punished stimulus contingencies reversed. The participant now had to learn to select the blue stimulus. **C** Trial-by-trial choice. Trial-by-trial averaged probability of selection of each stimulus. A sliding window with 5-trial width is used for smoothing. Overall, participants learned to make the correct selection for each of the phases and showed relatively rapid reversal. **D** Average choice probability. Distribution of choice probability averaged within acquisition (dark blue) and reversal (light blue) phases. In both phases, participants learnt to select 75% rewarded option but do significantly less well during the reversal phase. **E** Feedback sensitivity. The degree to which people repeated a choice was modulated by the valence of the previous outcome for that choice: People were more likely to reselect a stimulus (‘stay’) after it had been rewarded than after it was punished. This effect was weaker during the reversal phase (less stay after a win, more stay after a loss), in line with slower learning during reversal. Note that the intercept in this plot was chance level stay (1/3), even after a loss people were more likely to stay than chance (simple effect of pStay following loss: F(1,99) = 77.1; p < 0.0001, following win F(1,99) = 1603.4; p < 0.0001), reflecting a relatively small impact of a single (negative) outcome and thus slow learning

The probabilistic reversal learning task was the last task participants completed following capsule intake, at *t* = 186.1 (7.9) min, mean (st.d.). This task was preceded by 5 other tasks published elsewhere (Swart et al. 2017; Froböse et al. 2018; Cook et al. 2019) (Fig. 1). Both test days lasted approximately 4.5 h, which participants started at the same time of day (maximum difference of 45 min). Blood pressure, mood, and potential medical symptoms were monitored three times daily: before capsule intake (*t* =  − 5.3 (1.7) min), directly prior to start of the task battery (*t* = 47.4 (7.6)), and after finishing the task battery (*t* = 190.9 (7.9)). Mood and medical symptom ratings are described in supplemental methods 4. Participants were instructed to abstain from alcohol and recreational drugs 24 h prior to testing and from smoking and drinking coffee on testing days. Participants completed self-report questionnaires at home between test days. Upon completion of the study, participants received a monetary reimbursement or study credits for participation. The study was in line with the local ethical guidelines approved by the local ethics committee (CMO/METC Arnhem Nijmegen: protocol NL47166.091.13), preregistered (trial register NTR4653, http://www.trialregister.nl/trialreg/admin/rctview.asp?TC=4653), and in accordance with the Helsinki Declaration of 1975.

Participants were allocated pseudo-randomly to the intervention order (placebo vs methylphenidate first) in a double-blind, cross-over design. For details on the randomisation procedure, see supplemental methods 5.

### Participants

As specified in the preregistration, we planned to test a sample of 100 participants. To this end, 176 healthy, young adults were recruited via flyers around the campus and the digital participant pool of the Radboud University, Nijmegen. All participants were native Dutch speakers and provided written informed consent to participate. Exclusion criteria comprised a history of psychiatric, neurological, or endocrine disorders (see supplemental methods 3 for a complete overview of the exclusion criteria). One hundred six participants who met the inclusion criteria were included in this study to reach the planned 100 participants: Data from four participants could not be collected on day 2 due to medical reasons (mild arrhythmia: *n* = 1, elevated heart rate and nausea: *n* = 1) and drop-out (*n* = 2). These were replaced. A further 2 participants were replaced, who had difficulty swallowing the capsules and for whom the capsule content was suspended in water on both testing days. These 2 participants were included in the final analyses, as we verified that their inclusion/exclusion did not affect the results. Thus, the final analyses include 102 adult participants (aged 18–28 years, mean = 21.5, st.d. = 2.3, 51 women, 81 right-handed), where 50 participants received methylphenidate on the first testing session. Additional demographic information and results from baseline neuropsychological assessment and self-report questionnaires of included participants are reported in supplemental methods 6.

### Probabilistic reversal learning task

Participants performed a probabilistic reversal learning (PRL) task with three choice options in each trial. This task is an adjusted version of den Ouden et al. (2013), which consisted of 2 cues that were either predominantly rewarded during acquisition and predominantly punished during reversal (stimulus ‘*R-P’*) or the reverse (stimulus ‘*P-R’*). We here added a neutral stimulus with 50–50% contingencies throughout the task (stimulus ‘*N–N’*). The rationale for the addition of this neutral stimulus was to allow us to dissociate between two putative causes of perseverative behaviour. On the one hand, perseveration may result an inability to stop choosing a previously rewarded stimulus, so true ‘perseveration’ of the previously rewarded response. On the other hand, it may result from punishment avoidance, i.e. an in inability to approach a previously punished stimulus. Introducing a neutral stimulus makes differential predictions for these two scenarios. In case of the former, perseverative behaviour would manifest as continued selection of the previously rewarded response. However, in case of punishment avoidance, following reversal, the participant is able to unlearn the previously rewarded response but would fail to learn to select the previously punished response and will now preferentially select the neutral outcome.

On each of 80 trials, three visual stimuli {*R-P, P-R, N–N*} were represented in three out of four pseudo-randomly selected locations (left, right, top, or bottom; Fig. 1B). Participants chose one of the stimuli with a mouse click and subsequently received feedback. There was no time limit for responses. Feedback was either a reward (green, happy emotion) or punishment (red, sad emotion*)*. To maximise reward, participants had to learn by trial-and-error to choose the mostly rewarded stimulus. During the acquisition phase, stimulus *R-P* (defined as the first stimulus that was chosen by the participant) gives reward/punishment with contingencies of 75:25%, while P-R results in reward/punishment with the opposite (25:75%) contingency ratio. After 40 trials, these reinforcement contingencies reversed. Thus, the task consisted of an acquisition and a reversal phase. The third stimulus *N–N* is ‘neutral’, as its selection results in 50:50 ratio reward/punishment throughout the task. For information on the generation of the feedback sequence and full task instructions, see supplemental methods 1 and 2.

### Behavioural data analysis

#### Data quality assessment

A priori we decided to exclude any participant who selected the same stimulus the entire experiment as they likely did not understand the task (cf. den Ouden et al. 2013). However, this did not happen in the current sample. Trials with RT faster than 200 ms likely reflect responses that were not based on a deliberate choice between the stimuli. We confirm that results do not change whether these trials are included (main article) or excluded (supplemental results & discussion 3).

#### Choice accuracy

The statistics software SPSS (version 25) was used to analyse the behavioural choices. The probability of choosing each stimulus was calculated separately for the acquisition and reversal phases. To assess the putative mechanisms that may drive reduced performance following reversal, we contrasted two measures of performance accuracy: (i) the probability of selecting the 75% rewarded stimulus (pReward) and (ii) the probability of avoiding the 75% punished stimulus (1-pPunish or pAvoidPunish). To ensure that the intercept of this analysis was interpretable, we corrected scores for chance performance (i.e. subtracted 1/3). The basic design of this analysis was a 2 × 2 factorial ANOVA with factors Phase (acquisition/reversal) and Valence (pReward/pAvoidPunish). Note that these two measures are rendered (relatively) independent by inclusion of the neutral stimulus, which is the implicit baseline. The intercept of this ANOVA indexes the ability to learn to select the rewarded stimulus and avoid the punished stimulus. A main effect of Phase can capture the relative reduction of performance in the reversal phase. Most importantly, an interaction of Valence × Phase assesses whether there is a difference in the degree to which people fail to ‘let go’ of the previously rewarded stimulus (reduced pAvoidPunish selection during reversal) or the degree to which they fail to approach the previously punished stimulus (reduced pReward during reversal).

To assess the effect of methylphenidate on performance, this basic design was extended with the factor Drug, resulting in a three-way repeated measure ANOVA with Valence (pReward, pAvoidPunish), Phase (acquisition, reversal), and Drug (methylphenidate, placebo) as within-subject factors. Furthermore, listening span total score and Barratt impulsiveness score were added as covariates of interest. For reporting significant results, the Huyn-Feldt correction was used when significant non-sphericity was detected.

Any significant interactions were broken down into their component simple effects to aid interpretation of the effects.

#### Control analyses of effects of no interest

To verify that our findings are not confounded by covariates (age and Nederlandse Leestest voor Volwassenen (NLV); Dutch adult reading test; a measure of verbal intelligence) and factors (gender, testing order) of no interest, we repeated the analyses above including these covariates and factors and confirmed that significant results remained significant and non-significant results remained non-significant. Furthermore, mood and medical symptom ratings were monitored for safety reasons. Control analyses regarding the mood and medical symptom ratings are reported in the supplemental results (supplemental results & discussion 1). Finally, for consistency with previous work (Chamberlain et al. 2006; den Ouden et al. 2013), we also assessed trial-by-trial behavioural adjustments following rewards and punishments (illustrated in Fig. 1E, details in supplemental results & discussion 2).

### Computational modelling

We employed a computational modelling approach to quantify and compare latent mechanisms underlying the task behaviour and particularly the effects of methylphenidate as a function of working memory capacity. For this, we augmented a previously established model of a simpler variant of this reversal learning task (den Ouden et al. 2013) and assessed effects of methylphenidate on the various parameters in this model. We defined a family of four ‘base’ models that could capture behaviour on this task, in a 2 × 2 model space. Briefly, models could either contain a monotonically decreasing learning rate (Experience Weighted Attraction: EWA model (den Ouden et al. 2013)) or a learning rate that was allowed to increase or decrease as a function of surprise (RL-Pearce-Hall hybrid model (Li et al. 2011; Piray et al. 2019b)). Additionally, both EWA and hybrid models were also tested with an extension where the value of unchosen options is ‘forgotten’ with a forgetting learning rate (Ito and Doya 2009). We first fitted and compared these 4 ‘base’ models across both drug and placebo sessions, to establish the best model independent of drug (cf. model fitting and comparison, below). We then tested variations of the winning ‘base’ model to assess which parameter was affected by methylphenidate. The equations for all four base models are described in detail in the supplemental materials 8. Here we only present the winning base model and methylphenidate extensions.

The winning Experience Weighted Attraction model (EWA) (Camerer and Ho 1999) is an extended version of a standard reinforcement learning (RL) model. We have previously shown that this model can capture (variability in) perseverative behaviour in a simpler version of the current paradigm (den Ouden et al. 2013). The key feature of this model is the so-called experience-weight parameter, which models the increasing impact of past experience on subsequent decisions. With increasing exposure to each stimulus, its experience-weight increases, resulting in a reluctance to update beliefs about this stimulus. This feature makes the EWA model particularly suitable for modelling reversal learning impairment, effectively embodying a learning rate that reduces over time, thus rendering behaviour less flexible. The EWA model is described by the following equations:

1 $$n_{c,t+1}=n_{c,t}\times\rho+1 V_{c,t+1}=({V}_{c,t}\times\varphi\times n_{c,t}+\lambda_t)/n_{c,t+1} V_{\sim c,t+1}=V_{\sim c,t}$$

where ${n}_{c,t}$ is the experience-weight of choice $c$ on trial $t$, which is updated on every trial, using the experience decay factor $\rho$. The expected value of choice $c$ on trial $t$, ${V}_{c,t}$, is updated by integrating the feedback, ${\lambda }_{t} \epsilon \{-\mathrm{1,1}\}$; the decay factor for previous payoffs (inverse learning rate), $\varphi$; and the experience-weight ${n}_{c,t}$. Initially, the effective learning rate for each choice is high, and by experiencing a choice, the experience-weight increases, resulting in a reluctance to update the stimulus value based on new outcomes.

In the current three choice option task, it is more likely that an option remains unchosen on consecutive trials. Thus, the value of an unchosen option may be ‘forgotten’. This is reflected by the fact that the winning model EWA + F was augmented with a forgetting rate ${\alpha }_{f}$:

2 $${V}_{\sim c,t+1}= {(1-{\alpha }_{f})V}_{\sim c,t}$$

For ${\alpha }_{f}=0$, the model is equivalent to the base EWA model (Eq. 1), and for more positive values, the value of the unselected option will converge to the initial value faster.

For all models, to select an action based on the computed values, a soft-max function was employed to calculate the probability of each choice.

3 $$p\left({c}_{t}=i\right)=\frac{\mathrm{exp}(\beta {V}_{c=i, t})}{\sum_{j}\mathrm{exp}(\beta {V}_{c=j,t})}$$

Here, $\beta$ is the inverse temperature parameter. The $j \epsilon \{\mathrm{1,2},3\}$ contains the possible actions.

We took this winning EWA + F base model as the basis to assess what mechanism drove the behavioural difference between placebo and methylphenidate sessions. For this, we built a collection of models where we allowed each parameter in turn to be estimated separately for the methylphenidate and placebo sessions (cf. Swart et al. 2017) and then assessed which of these models (or the winning base model) was the best explanation of our data.

### Model fitting and comparison

All models were fitted to the trial-by-trial choices of each participant using Hierarchical Bayesian Inference (HBI) for concurrent model comparison, parameter estimation, and inference at the population level (Piray et al. 2019a). This approach has important advantages for both parameter estimation and model comparison, as parameters estimated by the HBI show smaller errors compared to other methods, while model comparison by HBI is robust against outliers and is not biased towards overly simplistic or complex models. The winning model was selected based on the protected exceedance probability (Rigoux et al. 2014), and we also report model frequency. As described above, we report two sets of model comparison. First, we established the winning ‘base’ model where we fitted data across both sessions (methylphenidate and placebo) to establish the overall best model to describe the data. We then extended the winning base model such that we allowed each parameter in turn to be differentially estimated for methylphenidate and placebo. In the supplemental materials, we include two further control analyses to verify the assumption that methylphenidate affected only one parameter (c.f. supplemental results & discussion 5). For further details on model fitting and comparison, see supplemental methods 9.

### Model validation

Model comparison evaluates whether a winning model is better than other models using an estimation of model evidence, which evaluates goodness of fit relative to model complexity. However, a winning model is not necessarily a good model. A good model should be able to regenerate key features of the original data (Wilson and Collins 2019), which we assessed through simulations. Using each participant’s estimated parameters, 100 artificial agents are simulated playing the task, and their choices averaged to represent each individual participant’s behaviour. Trial-by-trial simulated data were re-analysed in order to assess whether they captured the key effects of interest.

### Parameter inference

Finally, we assessed the nature of the effect of methylphenidate in the winning model. We used a *t*-test to establish whether there was a significant difference in parameter estimates under methylphenidate vs placebo and correlated the drug-induced change in parameter estimates to the covariates of interest, working memory span, and trait impulsivity (see below), using a Spearman correlation. Last, we assessed whether methylphenidate-induced changes in parameter estimates predicted the methylphenidate-induced change in raw behaviour. For all parameter analyses, we extracted subject level parameters from the first, non-hierarchical, estimation step. This is to prevent bias (specifically one magnifying the difference between drug conditions) that could result from the hierarchical model fitting procedure (Piray et al. 2019a).

### Covariate analyses: working memory capacity and trait impulsivity

Two covariates, the listening span test total score (Daneman and Carpenter 1980; Salthouse and Babcock 1991) and Barratt impulsiveness scale (BIS-11; (Patton et al. 1995)) were included in the main analyses, as (preregistered) putative proxies of inter-individual variability in baseline dopamine function. These measures have been shown with PET to correlate positively to dopamine function (Cools et al. 2008; Landau et al. 2009) and have been shown to predict dopaminergic drug effects (Kimberg et al. 1997; Kimberg and D’Esposito 2003; Frank and Claus 2006; Cools and D’Esposito 2011; van der Schaaf et al. 2014). For detailed descriptions of these measures, see supplemental methods 7.

## Results

### Data quality assessment

All 102 healthy participants completed the probabilistic reversal learning task in two sessions and were included in the analysis. No participants chose the same response option throughout. Participants made very few responses with RT < 200 ms (mean (st.d.) 0.2 (0.6)% of trials, range 0–4%), and these trials were excluded for the basic analyses. For computational modelling, analyses were repeated with and without these trials.

### Behavioural analyses

#### Choice accuracy

Participants successfully learned the three-option PRL task. People overall learnt to select the rewarded and avoid the punished stimulus (Intercept: *F*(1,99) = 1515.1, *p* < 0.001, *η*^2^ = 0.94; Fig. 1C). However, choice accuracy was lower during the reversal phase than during the acquisition phase (Phase: *F*(1,99) = 19.7, *p* < 0.001, *η*^2^ = 0.17, c.f. Figure 1D). There was no evidence of a differential preference to either fail to select the previously punished stimulus or fail to ‘let go’ of the previously rewarded stimulus (Valence × Phase: *F*(1,99) = 0.3, *p* = 0.6, *η*^2^ = 0.003), indicating that there was no difference in the reversal phase in terms of the degree to which people stuck to the previously rewarded stimulus relative to the degree to which they avoided the previously punished stimulus.

**Fig. 2:**
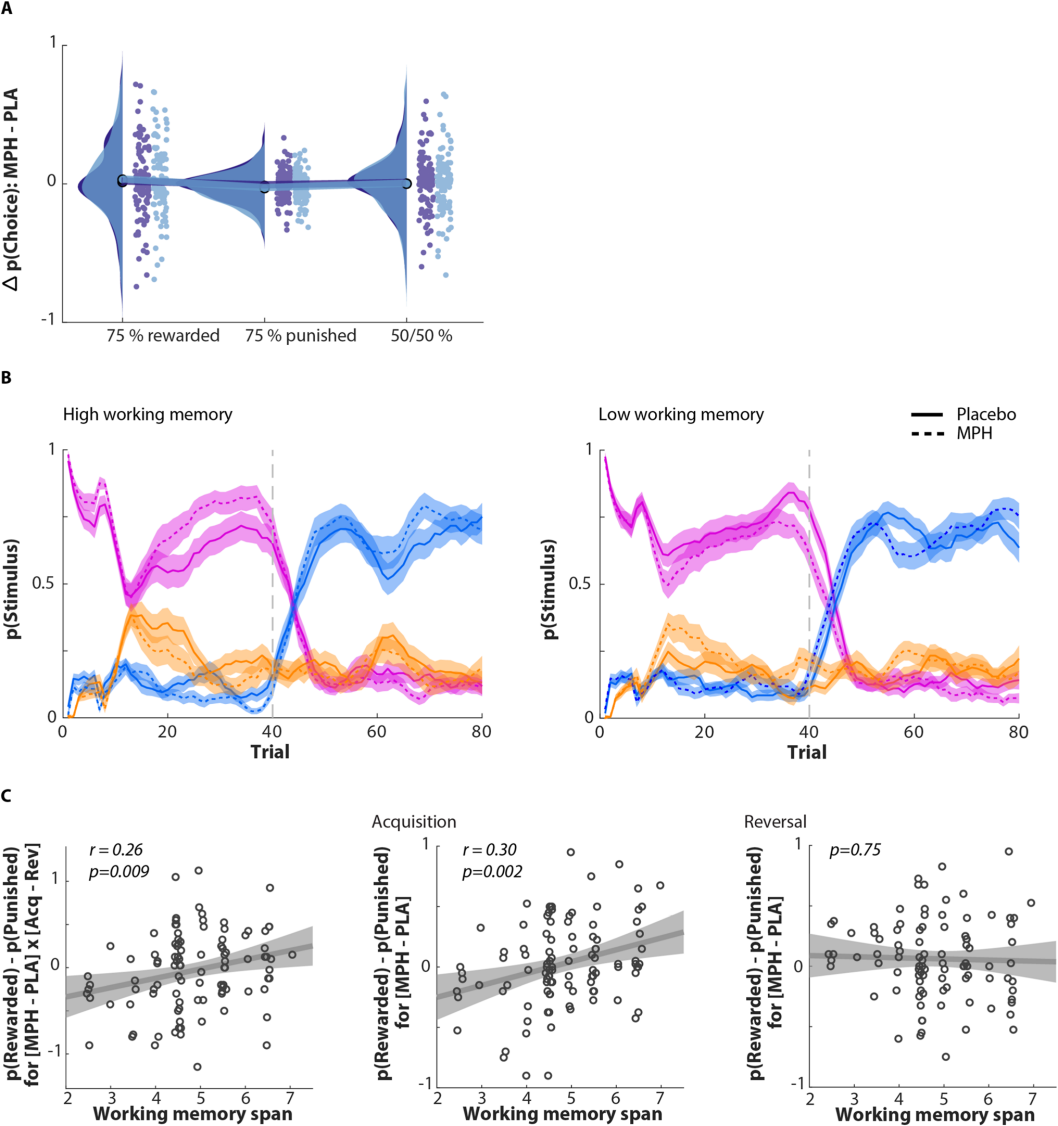
Effects of methylphenidate on reversal learning task performance. **A** No significant main effect of methylphenidate on average probability of stimulus selection. Distribution of difference of stimulus selection probability between two sessions (MPH-placebo) for acquisition and reversal phase is demonstrated in dark and light blue, respectively. Methylphenidate did not consistently affect either overall learning or differential learning during acquisition and reversal. **B** Trial-by-trial averaged probability of selection of each stimulus (median split based on WM span). Left panel: high WM group (n = 48), probability of ‘rewarded’ stimulus selection increased under methylphenidate (dash line) in comparison to placebo (solid line) during the acquisition phase. Right panel: low WM group (n = 54), probability of ‘rewarded’ stimulus selection decreased under methylphenidate in comparison to placebo during the acquisition phase. A sliding window with 5-trial width is used for smoothing. **C** Methylphenidate effects predicted by WM span. Methylphenidate increased the accuracy of selecting ‘rewarded’ option vs ‘punished’ option in acquisition phase more than reversal phase for high WM span participants yet decreased it for low WM span participants (r = 0.26, p = 0.009). By splitting up the MPH effect by phase factor, during the acquisition, middle panel, participants with high WM improved under methylphenidate (r = 0.30, p = 0.002), but during the reversal, right panel, there is no significant interaction

Methylphenidate did not consistently affect either overall performance (Drug: *F*(1,99) = 3.3, *p* = 0.074, *η*^2^ = 0.032), differential learning during acquisition and reversal (Phase × Drug: *F*(1,99) = 0.6, *p* = 0.43, *η*^2^ = 0.006), nor, importantly, reward-based perseveration relative to punishment-based avoidance (Phase × Valence × Drug: *F*(1,99) < 0.01, *p* = 0.96, *η*^2^ < 0.001). However, methylphenidate affected performance differentially during acquisition versus reversal as a function of WM span (Phase × Drug × WM span: *F*(1,99) = 7.1, *p* = 0.009, *η*^2^ = 0.067, Fig. 2C), which even weakly interacted with Valence (Phase × Valence × Drug × WM span: *F*(1,99) = 5.4, *p* = 0.022, *η*^2^ = 0.052). We followed up this interaction in two ways: First, to understand which factor drove this four-way interaction, we broke it down into simple effects for each of the factors (reported in detail in Supplemental results & discussion 3). The first key observation was that methylphenidate affected learning during the acquisition (*F*(1,99) = 9.8, *p* = 0.002), but surprisingly not during the reversal phase of the task (*F*(1,99) = 0.1, *p* = 0.75). During the acquisition phase, people with high working memory improved under methylphenidate, while people with low working memory performed more poorly (Fig. 2B). In contrast, during reversal, there was no interaction of performance with drug and working memory span. The second key observation, from post-hoc simple effects as a function of valence, was that methylphenidate strongly affected the ability to learn to select the rewarded stimulus (*F*(1,99) = 7.6, *p* = 0.007), and while effects of methylphenidate were in the same direction for the ability to learn to avoid the punished stimulus, these were significantly weaker and by themselves only a trend (*F*(1,99) = 3.7 *p* = 0.057). Interestingly, there was a significant effect for the probability to select the neutral stimulus (*F*(1,99) = 5.4 *p* = 0.022). Summarising, these results show that under methylphenidate, during the acquisition phase, people with high working memory were significantly likely to select the mostly rewarded stimulus while significantly less likely to select the neutral stimulus, leaving the punished stimulus not significantly affected. This suggests that in high working memory participants, methylphenidate aids to dissociate between the best and second best option.

Finally, there were some weak (trend) lower level interaction effects that were however qualified by the key higher-order interaction described above (Drug × WM span: *F*(1,99) = 3.6, *p* = 0.06, *η*^2^ = 0.036). There was no significant effect of BIS on performance, nor in interaction with methylphenidate (of interest: Phase × Drug × BIS: *F*(1,99) = 0.07, *p* = 0.8, *η*^2^ = 0.001; Phase × Valence × Drug × BIS: *F*(1,99) < 0.01, *p* = 0.9, *η*^2^ < 0.001; all other *p* > 0.1).

To control for potential confounding factors, we repeated this ANOVA by including the confound variables gender and test order (between subject factors) and age (covariate). Including these confound variables did not alter the significance of the observed effects although there was an effect of age (but this did not interact with the effect of interest; for details, see the supplemental results & discussion 1).

### Model comparison

**Fig. 3:**
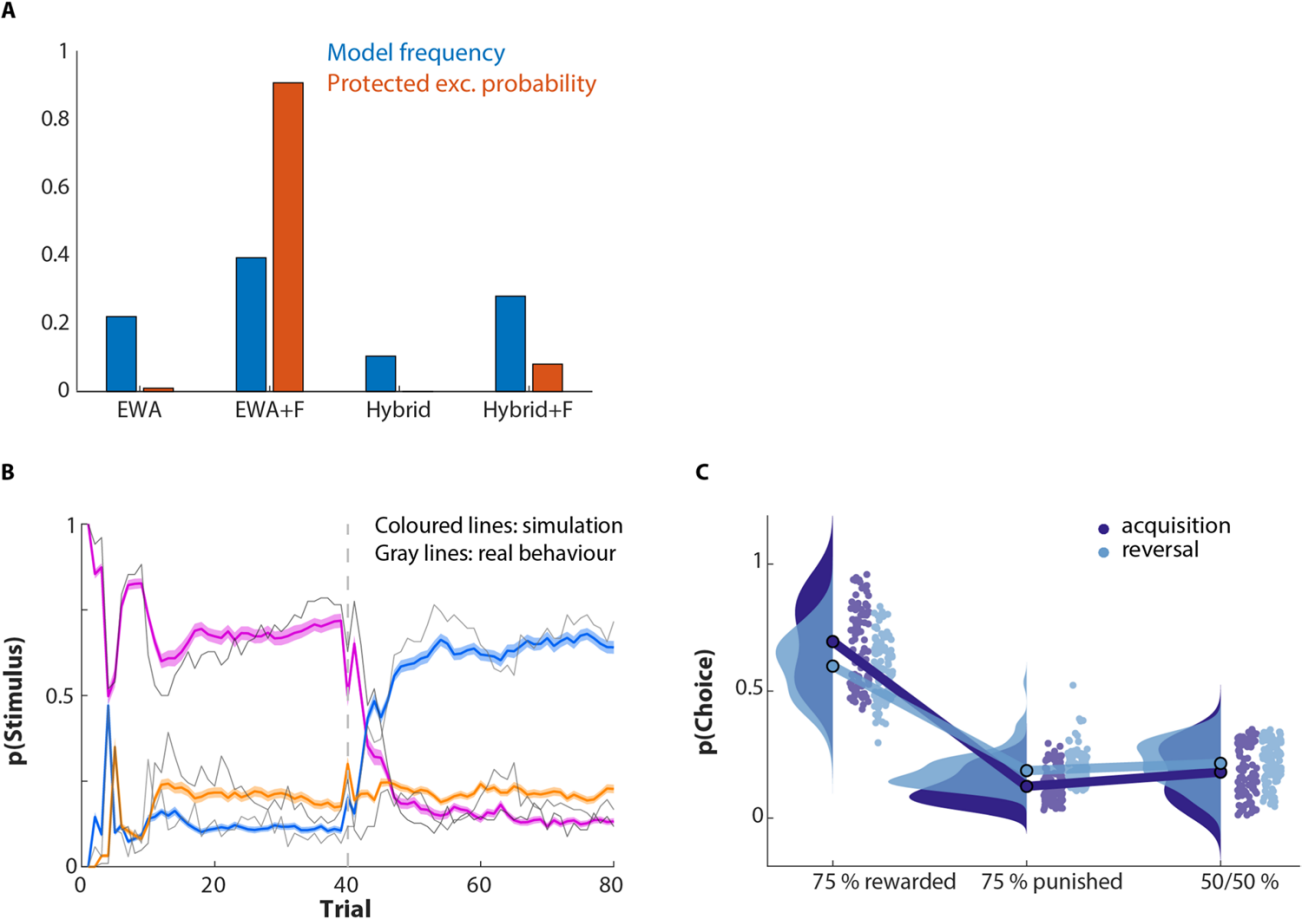
Model fitting and simulation result for PRL task modelling. **A** Model comparison on base models. Model frequency and protected exceedance probability indicate that the EWA + F model (EWA with forgetting rate for unchosen options) provides the best description of the data (PXP = 0.91). **B** Trial-by-Trial simulated choice. Model simulations of the winning base model verify that the EWA + F model captures the behavioural data (grey lines indicate average raw data). **C** Simulated average choice probability. Distribution of stimulus selection probability for acquisition and reversal phase is demonstrated in dark and light blue, respectively (compare with Fig. 1D). The simulated data for EWA + F model qualitatively and quantitatively replicate the participants’ behaviour and regenerate key features of the data

We compared our previously established EWA model to an extended version that included a forgetting factor, following from the extension of stimulus space (3-option design) along with the RL-Pearce-Hall hybrid models that allowed for adaptive, prediction error–based changes in learning rate. Model comparison showed convincing evidence in favour of the EWA + F model (protected exceedance probability: 0.91, Fig. 3A). See Table 1 and supplemental results & discussion 4 for parameter estimates for all four base models.

**Table 1:** Model evidence for base model and methylphenidate model families and parameter estimates for winning models in each family (see supplementary table 1 for full details for all models)

Base model family						
Model	Param.*	Constraint	Median	Range (25–75%)	pxp_i_	*p* (m_i_|data, m)
EWA					0.01	0.22
EWA + F					**0.91**	**0.40**
	$\varphi$	$0\le \varphi \le 1$	0.77	0.29–0.87		
	$\rho$	$0\le \rho \le 1$	0.63	0.27–0.83		
	$\beta$	$0\le \beta \le +\infty$	4.23	3.11–7.88		
	${\alpha }_{f}$	${0\le \alpha }_{f}\le 1$	0.35	0.02–0.68		
Hybrid					0.0	0.10
Hybrid + F					0.08	0.28
Methylphenidate model family						
EWA + F					0.02	0.25
EWA + F **+ **${\Delta }_{{\varvec{\rho}}}$					0	0.13
EWA + F **+ **${\Delta}_{\varphi}$					**0.98**	**0.42**
	${\varphi }_{Placebo}$	$0\le {\varphi }_{Placebo}\le 1$	0.70	0.26–0.86		
	${\varphi }_{MPH}$	$0\le {\varphi }_{MPH}\le 1$	0.70	0.33–0.84		
	$\rho$	$0\le \rho \le 1$	0.56	0.21–0.79		
	$\beta$	$0\le \beta \le +\infty$	4.46	3.13–7.94		
	${\alpha }_{f}$	${0\le \alpha }_{f}\le 1$	0.22	0.01–0.62		
EWA + F **+ **${\Delta}_{\alpha f}$					0	0.12
EWA + F **+ **$\triangle_\beta$					0	0.08

*A weakly informative Gaussian prior was used for all parameters (x∼N(μ,σ^2^) where the mean value$\mu =0$and the variance${\sigma }^{2}=10$). According to theoretical constraints of parameters, sigmoid or exponential transformations are applied.

**Fig. 4:**
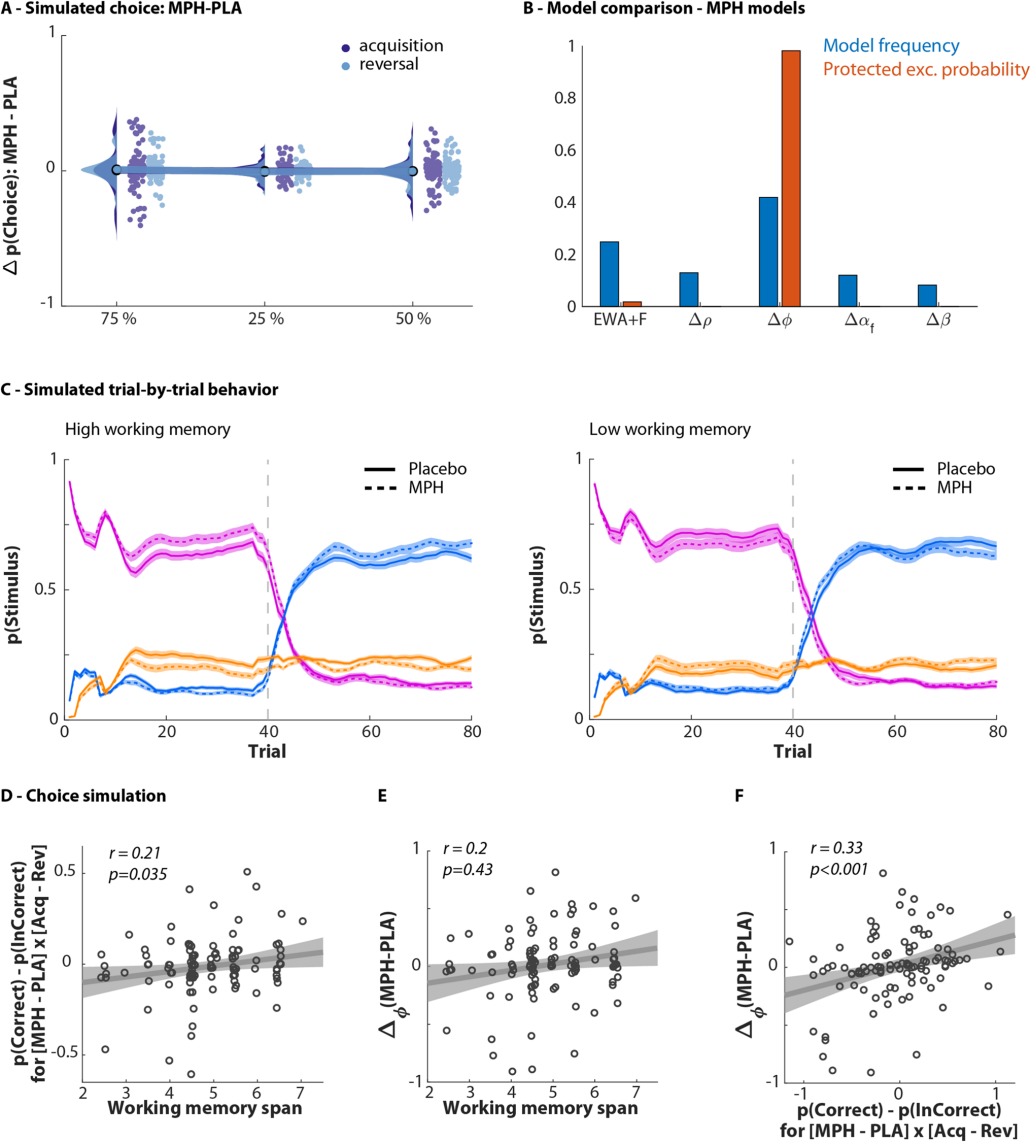
Modelling the effects of methylphenidate. **A** Simulated choice. Simulated data replicated observed behaviour for no main effect of methylphenidate (see Fig. 2A). **B** Model comparison on methylphenidate models. Model frequency and protected exceedance probability (PXP = 0.98) indicate that the EWA + F that allows for differential learning rates under methylphenidate and placebo best captures the data. Model validation: C-F. **C** Simulated trial-by-trial behaviour. Simulated data replicate observed behaviour for high WM and low WM participants (see Fig. 2B). **D** Choice simulation. The simulated data using winning model regenerate quantitative characteristics of data, particularly a positive effect of methylphenidate on performance for high WM participants (see Fig. 2C). **E** Methylphenidate changes inverse learning rate as a function of working memory span. The difference in inverse learning rates under methylphenidate vs placebo (${\Delta }_{{\varphi }}$) covaries with WM span (r = 0.2; p = 0.043). Methylphenidate increases ${\varphi }$ in high WM participants, where decreases it in low WM participants. **F** Methylphenidate-induced effect on raw performance scores. The methylphenidate-induced change in inverse learning rate (${\Delta }_{{\varphi }}$) is correlated with methylphenidate-induced change in raw performance (r = 0.33; p < 0.001)

To capture the effects of methylphenidate, we allowed each of the 4 parameters of this winning model to be affected by methylphenidate in turn, by fitting that parameter separately for methylphenidate and placebo sessions. We then compared all 4 ‘drug’ models plus the ‘baseline’ model without an effect of methylphenidate. Model comparison showed that the winning model allows methylphenidate to affect the inverse learning rate parameter $\varphi$ (protected exceedance probability: 0.98, see Fig. 4B). Parameter estimates for the methylphenidate models and model comparison statistics are reported in Table 1 and supplemental results & discussion 4. As described above, we assumed that methylphenidate affected only a single parameter (i.e. computational mechanism). We validate this assumption in the supplemental materials (see supplemental results & discussion 5).

### Model validation

Model simulation is essential to evaluate the model’s ability to regenerate key features of the data. In order to examine the reproducibility of the winning model, we generated data using the winning model. Our simulated data qualitatively and quantitatively replicate the participants’ behaviour (Figs. 3B–C and 4C–D). Again, in the simulated data, there is a significant interaction of Phase × Drug × WM span (*F*(1,99) = 5.1, *p* = 0.026, *η*^2^ = 0.049) (Fig. 4D). Furthermore, breaking down this interaction into simple effects by phase, our observations from raw data were replicated. Methylphenidate improved initial learning in high-WM span participants while reducing performance in low-WM span participants (*F*(1,99) = 5.1, *p* = 0.027, *η*^2^ = 0.049). Again, there was no significant Drug × WM span interaction during the reversal phase (*F*(1,99) = 1.7, *p* = 0.3, *η*^2^ = 0.011).

### Parameter inference

Model EWA + F, which allowed for a different value for the inverse learning rate $\varphi$ on vs off methylphenidate, was convincingly the best model. However, there was no significant difference between ${\varphi }_{Placebo}$ and ${\varphi }_{MPH}$ when assessed across all individuals (*t* =  − 0.4, *p* = 0.7). This indicates strong evidence for a *change* in $\varphi$ following drug intake, but the *sign* of this change was variable over participants. Indeed, mirroring the raw behavioural results, the difference between ${\varphi }_{Placebo}$ and ${\varphi }_{MPH}$ correlated with WM span (*r* = 0.20, *p* = 0.043, Fig. 4E). There was a strong correlation between methylphenidate-induced changes in inverse learning rate (${\Delta }_{\varphi }$) and the methylphenidate-induced change in raw performance metrics (*r* = 0.32, *p* < 0.001, Fig. 4F). Thus, methylphenidate administration increased performance in high working memory participants, which was captured by an increase in the inverse learning rate $\varphi$, thus effectively a decrease in learning rate. In contrast, methylphenidate administration decreased performance in low working memory participants, which was captured by a decrease in inverse learning rate $\varphi$. There was no correlation between the methylphenidate-induced changes in inverse learning rate (${\Delta }_{\varphi }$) and trait impulsivity (*r* =  − 0.02, *p* = 0.8).

### Optimal learning rate analysis

Compared with previous studies in which tasks were employed with two response options, we observed that performance in the acquisition phase was substantially worse, while performance during the reversal phase was much better in our current 3-option version (cf. den Ouden et al. 2013). Concomitant with this, values of $\varphi$ were much higher, i.e. learning rate was lower, in the current dataset than in our previous dataset. We therefore performed a supplemental analysis to compare the optimal learning rates across the two different versions of the task to assess whether this changed learning rate across paradigms was adaptive. In short, the 3-option version of the task had a lower optimal learning rate. This observation can be understood when realising that optimal performance on this probabilistic task required participants to dissociate a 50% reward option from a 75% reward. Integrating information over too short a time window (i.e. a high learning rate) would have made it more difficult to correctly dissociate between these two options. In line with this, the observed decrease in learning rate in high WM span participants under methylphenidate was adaptive, as the effective learning rates moved closer to the optimal learning rate. For details, see supplemental results & discussion 6.

## Discussion

This study aimed to uncover the causal role of dopamine in perseverative behaviour during probabilistic reversal learning. To this end, a large sample of participants (*n* = 102) performed a novel 3-choice PRL task both on and off catecholamine transporter blocker methylphenidate. Contrary to predictions, methylphenidate did not consistently affect perseverative behaviour in this novel task, as indexed by an absence of change in reversal performance. In contrast, methylphenidate altered performance in the acquisition phase, in a manner that depended on individual variability in working memory span. Specifically, methylphenidate increased the inverse learning rates in participants with a higher working memory span. In other words, in high WM individuals, methylphenidate reduced the degree to which values were updated following any single outcome, which made learning more robust in the probabilistic context of the task, thereby improving initial learning.

### Methylphenidate effects on value learning versus perseveration

We set out in this study to assess the computational mechanism by which catecholamine blockade affects reversal performance. Specifically, we asked whether reversal deficits were due to a failure to learn to approach a previously punished stimulus or to ‘let go’ of a previously rewarded stimulus. Perhaps a surprising finding in the current study is that acute catecholamine reuptake blockade did not affect reversal performance and perseveration at all. This is particularly surprising given the considerable literature concerning the role of dopamine in habitual actions (Daw et al. 2005; Everitt and Robbins 2005; Balleine and O’Doherty 2010) and perseverative behaviour (Cools et al. 2001; Rutledge et al. 2009), particularly direct findings of genetic variability in the *DAT1* genotype (den Ouden et al. 2013) and methylphenidate-induced reversal impairments (Clatworthy et al. 2009). While it is possible that this observed discrepancy between the previous literature and current study reflects a true non-replication in this large sample (*n* = 102, vs most previous studies *n* = 20–40), a perhaps more likely possibility is that, by introducing a neutral choice option, we have significantly altered the nature of the paradigm.

Indeed, as presented in the supplemental analyses, across participants, we observed that compared to the previously used 2-choice PRL paradigms, the learning rate was significantly lower. This reduction in effective learning rate provides an important clue as to why the observed effects in the current study are particularly obvious in the acquisition phase. By including the 50/50 choice option, the task was rendered much more difficult as optimal performance now required dissociation of two choice options (50/50 vs 75/25) that were much closer in terms of feedback than the previous (70/30 vs 30/70) dissociation that had to be learnt. To be able to make this dissociation, one needs to integrate information over a longer window, which is exactly what a reduced learning reflects. Indeed, this adjustment is adaptive, as optimal learning rates in the current paradigm are much lower than for the previous version of the task. In contrast, performance during the reversal phase was much more robust in the current paradigm.

This increased task difficulty may have had the unanticipated effect that this (difficult) initial learning was less robust across participants and became more sensitive to (e.g. drug) manipulations. We propose that dopamine affects both initial value learning, likely through ventral striatal prediction error like RL mechanisms that affect learning rate, but also affects longer-term ‘stamping in’ of responses and habit formation, through dorsal-striatal habit systems. Discrepancies in findings across studies, then, might reflect the relative sensitivity of various tasks tapping into these mechanisms. Thus, the current paradigm increased difficulty of initial learning but was also associated with less vulnerable reversal performance, while the reverse may be true for the 2-option task. It is unclear why performance during the reversal phase was so much better than in the 2-option task—perhaps the task structure was more obvious to participants. Regardless, the absence of methylphenidate effects on reversal performance therefore unfortunately did not allow us to further disentangle putative dopaminergic mechanisms of reward-based perseveration versus a failure to overcome learned avoidance.

### Mechanisms of baseline-dependent effects of methylphenidate

The finding that effects of methylphenidate on behaviour vary as a function of working memory capacity was consistent with our preregistered hypothesis. We posit two possible explanations for this effect. First, working memory span has been shown to correlate with striatal dopamine synthesis capacity (Cools et al. 2008; Landau et al. 2009). Given that methylphenidate acts by blocking the dopamine (and noradrenaline) transporter, it is likely that the effect of methylphenidate on catecholamine-dependent function is a function of dopamine synthesis capacity and subsequent release. While under placebo conditions, release and reuptake are in balance in both high and low synthesis capacity subjects, administration of methylphenidate could disturb this balance differentially. Specifically, if individuals with higher working memory capacity have higher release of dopamine (Cools et al. 2008; Landau et al. 2009), then methylphenidate might increase tonic levels of dopamine, paradoxically leading to reduced sensitivity to individual bursts and thus a reduced learning rate. In contrast, low working memory participants with low synthesis capacity may have very sensitive post-synaptic dopamine function, and blockade of transporters may increase the duration of post-synaptic impact of dopamine bursts, thereby effectively increasing the learning rate. A recent dopamine PET study indeed demonstrated disproportionate sensitivity of participants with low dopamine synthesis capacity to methylphenidate-related increases in reward impact on choice (Westbrook et al. 2020).

Alternatively, the interaction between methylphenidate effects on learning and baseline working memory capacity might reflect a modulation of interactions between working memory and reinforcement learning strategies (Collins et al. 2017). Specifically, Collins and Frank have recently established that within individuals, relative reliance on reinforcement learning versus working memory strategies varies with working memory load (Collins and Frank 2012). By analogy, we hypothesise that this balance in any given task may vary across individuals as a function of their working memory capacity. In short, if you have a lower span, you may shift sooner to RL strategies. Neurally, methylphenidate may act on striatal levels of dopamine, as suggested above, but may also affect frontal functioning, through blockade of noradrenaline transporters in the frontal cortex (Volkow et al. 2001, 2012; Arnsten and Dudley 2005; Berridge et al. 2006; Berridge and Devilbiss 2011; Kodama et al. 2017). The relative balance of the effect of methylphenidate on either striatal (putatively RL) mechanisms versus putative direct frontal modulation on working memory functioning may differ between individuals with high versus low baseline working memory capacity, explaining the differential effects observed.

A final speculation is that methylphenidate plays a role via its action on either dopamine or noradrenaline transmission by affecting our ability to optimise the learning rate given the volatility of the environment (Nassar et al. 2010; Muller et al. 2019). This hypothesis concurs with the results of our other experiment in the same individuals in which we employed a task explicitly designed to assess effects on learning as a function of the volatility of outcome contingencies (cf. Figure 3; Cook et al. 2019). In this learning task, methylphenidate adaptively lowered the learning rate in stable versus changeable environments. In the current experiment, the learning rate decrease in high capacity participants moved it closer to the optimal learning rate for current the task and was indeed associated with an increase in initial performance, due to a better ability to distinguish between the best (rewarded) and second-best (neutral) option. We do note that this ‘meta-learning’ interpretation should be taken with caution, because the current paradigm with its single reversal was not optimised to answer this question and the models in which we allowed the learning rate to fluctuate according to the size of the prediction errors did not perform better than the winning model in which the learning rate was only allowed to be reduced over time.

## Conclusion

The present study was set up to test the specific hypothesis, derived from our previous dopamine genetic study, that administration of methylphenidate would alter probabilistic reversal learning by changing the reliance on prior reward. To test this hypothesis, a novel reversal task was employed with three-choice options. Surprisingly, results revealed no effects on the reversal phase. However, an effect of methylphenidate surfaced already in the initial acquisition phase. In line with prior studies, this effect was not unidirectional across participants, but varied with individual differences in baseline working memory capacity: Methylphenidate improved performance and reduced the learning rate to a greater degree in participants with higher working memory capacity. We hypothesise that the increased demands for learning in this 3-option task brought to the surface an effect of methylphenidate on learning rather than on flexibility.

## Supplementary Information

Below is the link to the electronic supplementary material.

Supplementary file1 (DOCX 684 KB)

## Data Availability

The collected data and analysis scripts of the current study are available in the Donders Institute Data repository, https://data.donders.ru.nl/collections/di/dccn/DSC_3017031.02_887.
