# Supplementary material for: Effects of methylphenidate on reinforcement learning depend on working memory capacity
Source: Psychopharmacology (Berl). 2021 Oct 21;238(12):3569–84. doi: 10.1007/s00213-021-05974-w (PMC8629893; doi:10.1007/s00213-021-05974-w)
Supplement: Supplementary file 1 — Supplementary file1 (DOCX 684 KB) [file 213_2021_5974_MOESM1_ESM.docx]

Table of Contents

[Supplemental Methods 1](#_Toc77755658)

[1. Task instructions 1](#_Toc77755659)

[2. Task feedback sequence 2](#_Toc77755660)

[3. Overview of exclusion criteria 2](#_Toc77755661)

[4. Medical symptoms rating scale 3](#_Toc77755662)

[5. Randomisation procedure for drug allocation 3](#_Toc77755663)

[6. Demographic information 4](#_Toc77755664)

[7. Covariates of interest 5](#_Toc77755665)

[Working memory capacity 5](#_Toc77755666)

[Trait impulsivity 5](#_Toc77755667)

[8. Base model space 6](#_Toc77755668)

[9. Model fitting & comparison details 7](#_Toc77755669)

[Supplemental results & discussion 8](#_Toc77755670)

[1. Control analyses 8](#_Toc77755671)

[2. Win-Stay Lose-Shift analyses 9](#_Toc77755672)

[3. Post hoc interaction analyses to characterize main 4-way interaction 11](#_Toc77755673)

[4. Subject-level optimal parameter estimates for full model space 12](#_Toc77755674)

[5. Model space validation 13](#_Toc77755675)

[6. Performance comparison for 2 vs 3 choice option PRL task 15](#_Toc77755676)

[Optimal learning rate reduced in 3-choice PRL task 17](#_Toc77755677)

[References 18](#_Toc77755678)

# Supplemental Methods

## Task instructions

Below is an English translation of the full task instructions. Note that the task code (matlab, psychopy) and analysis code can be found at <https://github.com/denOudenLab> and https://data.donders.ru.nl/collections/di/dccn/DSC_3017031.02_887

*“On each go, three pictures will be presented. And you can select one picture by clicking on it with the mouse. The pictures differ in how often they are correct. You should choose the picture that tends to be correct most often, and you have to find out by trial and error which picture that is. At certain moments, the rules can change, so another picture is now correct most often. Then, switch your response to that picture. This can happen one or more times during the game. It is important to realize that the feedback you get ONLY depends on the picture that you chose. So it does not depend on the location of the picture, nor on your previous choices.”*

## Task feedback sequence

We generated pseudorandom fixed sequences for each cue (R-P, P-R, N-N) and task phase (acquisition, reversal), i.e. 3x2 sequences of 40 trials each. Each sequence consisted of a series of outcomes that followed the relevant reward contingencies. Every time a specific stimulus was chosen, the next outcome in this feedback sequence was presented. In other words, if the initially rewarded stimulus was chosen 30 times during the acquisition phase, the first 30 outcomes from the associated feedback sequence were presented. We used this set-up rather than a fixed outcome sequence as used in previous studies (e.g. (den Ouden et al. 2013)) to ensure that participants would experience the correct cue-outcome contingencies (so 75:25:50 for R-P:P-R:N-N prior to reversal, and 25:75:50 post reversal), regardless of their sequence of choices. This was particularly important for the currently used 3 choice option task, where, unlike the 2-choice option, it was not possible to infer what the outcome would have been had another option been chosen.

Stimulus notation:

R-P: 75% rewarded during acquisition, 75% punished during reversal

P-R: 75% punished during acquisition, 75% rewarded during reversal

N-N: 50/50 % reward / punishment throughout

## Overview of exclusion criteria

- (History of) psychiatric treatment
- (History of) neurological treatment
- (History of) endocrine treatment
- (History of) autonomic failure (e.g., vasovagal reflex syncope).
- (History of) clinically significant hepatic, cardiac, obstructive respiratory, renal, cerebrovascular, metabolic or pulmonary disease
- Family history of sudden death or ventricular arrhythmia
- (History of) epilepsy
- (History of) drug dependence (opiate, LSD, (meth)amphetamine, cocaine, solvents, or barbiturate) or alcohol dependence
- Suicidality
- Abnormal hearing or (uncorrected) vision.
- Use of MAO inhibitor, anaesthetic, anti-depressant or antipsychotic drugs within the week prior to the start of the study.
- Use of psychotropic medication, or of recreational drugs over a period of 24 hours prior to each test session, and use of alcohol within the last 24 hours before each measurement.
- Regular use of corticosteroids.
- Uncontrolled hypertension, defined as diastolic blood pressure at rest > 95 mmHg or systolic blood pressure at rest > 180 mmHg
- Hypotension, defined as diastolic blood pressure < 50 mm Hg or systolic < 95 mm Hg or resting pulse rate < 45 beats/min
- Diabetes
- Family history of schizophrenia, bipolar disorder or major depressive disorder
- Irregular sleep/wake rhythm (e.g., regular nightshifts or cross timeline travel).
- Possible pregnancy or breastfeeding
- Lactose intolerance (placebo pill is a lactose product)

## Medical symptoms rating scale

1. No headache Strong headache
2. No muscle pain Strong muscle pain
3. No dry mouth Very dry mouth
4. Not dizzy Very dizzy
5. No abdominal pain Strong abdominal pain
6. No joint pain Strong joint pain
7. No trouble breathing Trouble breathing
8. No throat pain Strong throat pain
9. No chest pain Strong chest pain
10. No eye problems Strong eye problems

## Randomisation procedure for drug allocation

The pseudo-random allocation sequence was generated by the Radboud University Medical Centre pharmacy, Clinical Trials Unit. Random allocation was implemented using sequentially numbered containers (e.g. ‘participant 1, day 1’), prepared and delivered by the pharmacy and administered by the experimenters. Thus, experimenters and participants were blinded to assignment to intervention. Randomisation order was balanced between genders by using allocation sequence numbers 1-50 for men, and 51-100 for women. Upon drop-out of any participant, these were replaced by the pharmacy to maintain the randomisation sequence.

## Demographic information

Demographic information, and results from baseline neuropsychological assessment and self-report questionnaires of included participants are reported in *Table S1.*

| **N=102** | **Characteristic** | **Measure** | **mean (st.d.)** | **min-max** | **range*** |
| --- | --- | --- | --- | --- | --- |
| **Demographics** | Age | Years | 21.5 (2.3) | 18-28 | - |
|  | Gender | Men/women (number) | 51/51 | - | - |
| **Experimental information** | Order | Placebo first / MPH first (number) | 52/50 | - | - |
|  | Mean delay MPH to task start | Minutes | 186.1 (7.9) | - | - |
| **Neuropsychological assessment** | Verbal intelligence | NLV | 93.5 (7.7) | 75 - 114 | 55 – 145 |
|  | Working memory capacity | Listening span: total span | 4.8 (1.0) | 2.5 - 7 | 0 – 7 |
|  |  | Digit span**  Forward  Backward | 16.7(3.6) | 10 - 26 | 0 – 28 |
|  |  |  | 14.4(3.1) | 8 – 23 | 0 – 28 |
| **Self-report**  **Questionnaires** | Impulsivity | BIS-11: total score | 61.8 (8.5) | 43 - 93 | 30 – 120 |
|  | Need for Cognition | NCS | 63.4 (10.5) | 38 - 82 | 18 – 90 |
|  | Depressive symptoms | BDI | 3.5 (3.8) | 0 – 21 | 0 – 63 |
|  | Behavioral activation | BAS: total score | 23.3 (4.0) | 15 - 34 | 13 – 52 |
|  | Behavioral inhibition | BIS | 16.3 (3.5) | 7 – 23 | 7 – 28 |
|  | Anxiety symptoms | STAI | 32.5 (6.9) | 23 - 55 | 20 – 80 |
|  | Social support | MDSPSS: total | 70.4 (9.5) | 43 - 84 | 12 – 84 |
|  | Social status | BSMSS: total | 47.9 (12.6) | 14.5- 66 | 8 – 66 |
|  | Social dominance | SADQ: social | 4.1 (0.8) | 2.1 - 5.9 | 1 – 7 |
|  | Aggressive dominance | SADQ: aggressive | 2.6 (0.6) | 1.3 - 4.6 | 1 – 7 |

***Table S1 Demographic and background characteristics of participants included in the analysis.***

*Demographic and background characteristics of participants included in the analysis. Questionnaires included the Beck Depression Inventory (BDI;* (Beck et al. 1996)*), Behavioral Inhibition Scale/Behavioral Activation Scale (BIS/BAS;* (Carver and White 1994)*), Barratt Impulsiveness Scale (BIS-11;* (Patton et al. 1995)*), Need for Cognition Scale (BCS;* (Cacioppo et al. 1984)*), Spielberger Trait Anxiety Inventory (STAI;* (Spielberger et al. 1983)*), Multidimensional Scale of Perceived Social Support (MDSPSS;* (Zimet et al. 1988)*), Social and Aggressive Dominance Questionnaire (SADQ;* (Kalma et al. 1993)*) and Barratt Simplified Measure of Social Status (BSMSS;* (Barratt 2006)*). Reported scores are comparable with observations in healthy populations in earlier reports. Listening span,* (Salthouse and Babcock 1991)*; Digit span,* (van der Schaaf et al. 2014)*: FW mean = 8.5; BW mean = 7.9; BIS-II,* (Buckholtz et al. 2010)*: mean = 59.5; NCS,* (Westbrook et al. 2013)*: mean = 64.5; BDI,* (Schulte-Van Maaren et al. 2013)*: mean = 3.7; BIS/BAS,* (Franken et al. 2005)*: mean BIS = 13.8, mean BAS = 24.5; STAI,* (De Weerd et al. 2001)*: mean ≈ 34; MDSPSS,* (Canty-Mitchell and Zimet 2000)*: mean = 5.5; BSMSS,* (Cook et al. 2014)*: mean = 49.0, 42.6; SADQ,* (Cook et al. 2014)*: social mean = 4.0, 3.9; aggressive mean = 2.9, 2.7. The verbal IQ estimate (NLV) seems low in this sample (relative to* (van der Schaaf et al. 2014)*: mean = 101). However, we tested a student population and we expect this value to be low due to the outdated character of the test (1991), not accommodating changes in language use, particularly integration of English words in the Dutch language.*

**Note: range reflects the possible range of scores on the questionnaires, whereas min - max and mean (st.d.) reflect the participant data.*

*** scores represent an average across two testing days.*

## Covariates of interest

### Working memory capacity

The listening span test (Daneman and Carpenter 1980; Salthouse and Babcock 1991) was administered at the beginning of the second test session, i.e. prior to drug administration, to obtain an estimate of participants’ ‘baseline’ working memory capacity, as a (preregistered) putative proxy of baseline dopamine synthesis capacity. Total listening span has been shown to correlate positively with dopamine synthesis capacity (Cools et al. 2008; Landau et al. 2009), and has been shown to predict dopaminergic drug effects (Kimberg et al. 1997; Kimberg and D’Esposito 2003; Frank and Claus 2006; Cools and D’Esposito 2011; van der Schaaf et al. 2014).

During this test, participants listened to pre-recorded sentences and were given two tasks: They answered simple written multiple-choice questions about the content while remembering the last word of each sentence for later recall. The number of sentences on each trial (i.e. the span) increased up to 7 over the course of the task. Three series of each span were conducted. The trial was coded as successful if the answers to the multiple choice questions were correct and if all last words were remembered and reported in the correct order. Based on participants’ performance a listening span was calculated ranging from 0 to a maximum of 7. The highest level for which two out of the three series were correctly remembered comprised the *basic span*. Half a point was added if one series of the following span was correctly completed, resulting in the measure of *total span*.

### Trait impulsivity

Trait impulsivity was assessed with the Barratt Impulsiveness Scale (BIS-11; (Patton et al. 1995)), as a second (preregistered) putative proxy of baseline dopamine function for predicting effects of methylphenidate. The BIS-11 is a self-report questionnaire, consisting of 30 statements tapping in common (non)impulsive behaviours and preferences, that participants rate on a 4-point Likert scale (“never” to “almost always”). Scores have been found to be associated with dopamine D2/D3 receptor availability in the midbrain, and enhanced dopamine release in the striatum (Lee et al. 2009; Buckholtz et al. 2010; Reeves et al. 2012; Kim et al. 2014) and has been shown to predict effects of methylphenidate on learning (Clatworthy et al. 2009). Three participants did not complete one (n=2) or two (n=1) items of the questionnaire. For these items, we used the mean answer across all participants. Other questionnaire data were acquired to establish successful randomisation, and are presented in Supplemental methods 6.

## Base model space

Model space comprised four models. The first model known as the Experience-Weighted Attraction model (EWA) (Camerer and Ho 1999), and is an extended version of a standard reinforcement learning (RL) model. We have previously shown that this model can capture (variability in) perseverative behaviour in a simpler version of the current paradigm (den Ouden et al. 2013). The key feature of this model is the so-called experience-weight parameter, which models the increasing impact of past experience on subsequent decisions. With increasing exposure to each stimulus, its experience-weight increases, resulting in a reluctance to update beliefs about this stimulus. This feature makes the EWA model particularly suitable for modelling reversal learning impairment, effectively embodying a learning rate that reduces over time thus rendering behavior less flexible. The EWA model is described by the following equations:

|  | $n_{c,t+1}= n_{c,t}\times\rho+1$ $V_{c,t+1}= {(V}_{c,t}\times\varphi\times n_{c,t}+\lambda_{t})/ n_{c,t+1}$ $V_{\sim c,t+1}= V_{\sim c,t}$ | () |
| --- | --- | --- |

where $n_{c,t}$ is the experience-weight of choice $c$ on trial $t$, which is updated on every trial, using the experience decay factor $\rho$. The expected value of choice $c$ on trial $t$, $V_{c,t}$, is updated by integrating the feedback, $\lambda_{t} \epsilon\{-1,1\}$, the decay factor for previous payoffs (inverse learning rate), $\varphi$, and the experience-weight $n_{c,t}$. Initially, the effective learning rate for each choice is high ($ELR=1/{(1+\varphi)}$), by increasing the experience-weight to its asymptote point ($n_{. ,\infty}=1/{(1-\rho)}$), the learning rate will decrease and converge to ${ELR}_{c,\infty}=1/{\left( 1\times\varphi\times n_{c,\infty} \right)={(1-\rho)}/{(1-\rho+\varphi)}}$. Therefore, by experiencing a choice, the experience-weight increases, resulting in a reluctance to update the stimulus value based on new outcomes. The value of unchosen options $V_{\sim c,t}$, remains unchanged.

In second model, EWA is augmented with a forgetting rate. The current version of this task has three choice options. Therefore, in comparison with the two-option task, it is more likely that an option remains unchosen on consecutive trials. Thus, the value of an unchosen option may be ‘forgotten’. In this EWA+F model, the value of unchosen options is ‘forgotten’ by pulling these to their initial value (Ito and Doya 2009):

|  | $V_{\sim c,t+1}= {(1-\alpha_{f})V}_{\sim c,t}$ | () |
| --- | --- | --- |

here, $\alpha_{f}$ is the forgetting rate. For $\alpha_{f}=0$, the model is equivalent to the base EWA model (Eq. 1) and for more positive values, the value of the unselected option will converge to the initial value faster.

In the EWA models, the learning rate can only decrease over time (or remain constant). However, it would be adaptive to decrease learning rates in periods of stability and increase learning rates in periods of change. Indeed previous studies have been shown that people are able to adjust their learning rate according to the volatility of the environment (Behrens et al. 2007). It is unclear, however, whether people adjust their learning rate when there is only a single reversal, or whether this takes multiple reversals to ‘meta-learn’ that changes take place. Furthermore, even if people do adjust their learning rate, it is unclear if we can reliably quantify this with only a single reversal. These are, however, empirical questions, and thus to assess such adaptive changing of the learning rate, Hybrid comprised models (Li et al. 2011; Piray et al. 2019b) where the learning rate is a function of the absolute value of previous prediction error (surprise signal). Following reversal, continued choice of the initially rewarded stimulus will lead to a series of prediction errors, thus increasing the learning rate and allowing for faster reversal. In this hybrid model, values are updated as follows:

|  | $\delta_{t}=o_{t}-V_{c,t}$ $\alpha_{t}=\kappa A_{t}$ $V_{c,t+1}= V_{c,t}+\alpha_{t}\delta_{t}$ $V_{\sim c,t+1}= V_{\sim c,t}$ $A_{t+1}= \eta\left\vert\delta_{t} \right\vert+(1-\eta)A_{t}$ | (3) |
| --- | --- | --- |

where $\kappa$ is scale of learning rate and $\eta$ determines the step size for updating associability of the cue. On each trial, the learning rate $\alpha_{t}$, depends on associability $A_{t}$, which itself is updated based on absolute prediction error from the past trials, but not the current one. Therefore, $\delta_{t}$ is not double counted in the value update, so this makes $\delta_{t}$ and $A_{t}$ relatively uncorrelated. Note that, the value of unchosen options $V_{\sim c,t}$, remains unchanged.

Finally, in Hybrid+F model, Hybrid model was augmented with a forgetting rate, where unchosen options are updated using Eq. 2.

For all models, to select an action based on the computed values, a soft-max function was employed to calculate the probability of each choice.

|  | $p\left( c_{t}=i \right)=\frac{exp(\beta V_{c=i, t})}{\sum_{j} exp(\beta V_{c=j,t})}$ | (4) |
| --- | --- | --- |

here, $\beta$ is the inverse temperature parameter. The $j \epsilon\{1,2,3\}$ contains the possible actions.

We first fitted and compared these 4 ‘base’ models across both drug and placebo sessions, to establish the best model independent of drug (cf. model fitting and comparison, below).

## Model fitting & comparison details

All models were fitted to the trial-by-trial choices of each participant using Hierarchical Bayesian Inference (HBI) (Piray et al. 2019a). The HBI procedure employs a full hierarchical Bayesian approach for concurrent parameter estimation and random effect model comparison. This method uses variational Bayes (Jordan et al. 1998), an extended version of expectation-maximization (Dempster et al. 1977), for estimating individual and group-level parameters using an iterative algorithm. The random effects approach assumes that different models might be responsible for generating data for different participants. Therefore, participants have different influences on updating group-level parameters (mean and variance), where better-fitted participants have more weight in updating the group-level parameters. This weight is based on a so-called responsibility measure, quantifying the probability that each model is responsible for generating data for a particular participant. For parameter estimation, a wide Gaussian prior (mean 0; variance 10) was assumed for all parameters. Then, according to theoretical constraints of parameters, $\rho,\varphi,\alpha_{f},\kappa,\eta\epsilon[0,1]$ and $\beta\epsilon[0,+\infty)$, we apply sigmoid and exponential transformations, respectively.

For model comparison, the best model was determined using random effects model comparison (Stephan et al. 2009; Rigoux et al. 2014), using a Laplace approximation (Kass, R and Raftery, A 1995; Daw 2011) of model evidence for each individual participant in order to compute the group evidence. The group evidence quantifies how well each model fits to the data across all participants, penalized for model complexity. The winning model was selected based on the protected exceedance probability (Rigoux et al. 2014) and we also report model frequency. We report two sets of model comparison. First we established the winning ‘base’ model where we fitted data across both sessions (methylphenidate and placebo) to establish the overall best model to describe the data. We then extended the winning base model such that we allowed each parameter in turn to be differentially estimated for methylphenidate and placebo. This approach turns the question of the effects of methylphenidate on any of the parameters into a model comparison question. The great advantage of this approach rather than just fitting all parameter estimates on vs. off is twofold. First, it allows us to establish whether there is an effect of methylphenidate, independent of whether that effect is consistent in ‘sign’ (positive or negative) across individuals. This is particularly suitable when assessing dopaminergic drug effects which are well-known to vary greatly over participants(Cools and D’Esposito 2011). Second, it is more parsimonious than allowing all parameters to vary freely with methylphenidate, using only 1 extra parameter per model, and thus reducing the risk of overfitting the data, which in the current dataset with only 80 trials per session is relevant. In the supplemental result & discussion 5, we include two further control analyses where verify the assumption that methylphenidate affected only one parameter.

# Supplemental results & discussion

## Control analyses

Age, gender, test order and NLV (Nederlandse Leestest voor Volwassenen – Dutch Adult Reading Test, a measure of verbal intelligence) are potential factors that may explain variance in behaviour. We therefore included these covariates (age, NLV) and factors (gender, test order) in the main analysis, primarily to assess whether inclusion of these variables affected our main findings. The interaction of Phase x Drug x WM span remained significant F(1,94)=7.26, p=.008, η2=.072), as did the less significant four-way interaction of Valence x Phase x Drug x WM span (F(1,94)=4.7, p=.034, η2=.047).

We observed some interactions of age with drug, which we report here for completeness (Valence x Phase x Drug x Age: F(1,94)=5.67, p=.02, η^2^=.052; Phase x Drug x Age: F(1,94)=4.06, p=.047, η^2^=.041). However, we are reluctant to interpret these effects given that none of these interactions survive multiple comparison correction, and that the age range of our sample was narrow (mean (st.d.) = 21.5 (2.3); min-max = 18-28). Importantly, there is no significant correlation between age and WM span (r=.08, p =.37). Again for completeness, there were significant interactions of Phase x Gender (F(1,94)=4.09, p=.04, η^2^=.042), Valence x Phase x Gender x Test Order (F(1,94)=4.17, p=.04, η^2^=.043), and trends of Valence x Drug x Test Order (F(1,94)=2.9, p=.092, η^2^=.030)); Valence x Drug x BIS (F(1,94)= 2.9, p=.092, η^2^=.030)). There were no further significant or trend effects of these nuisance variables (F<2.2, p>.14).

Finally, mood ratings, heart rate and blood pressure were monitored for safety reasons i) before capsule intake, ii) upon start task battery, and iii) upon completion of the task battery. The mood ratings consisted of the Positive and Negative Affect Scale (PANAS; (Watson et al. 1988)) and the Bond and Lader Visual Analogues Scales (calmness, contentedness, alertness; (Bond and Lader 1974)), as well as a medical Visual Analogues Scale. We assessed whether methylphenidate affected mood and medical symptoms. For this control analysis, we performed a repeated measures MANOVA using Pillai’s trace with the within subject factors Time (prior to drug intake; prior to the first task; at the end of testing, see *Figure 1.A*) and Drug (methylphenidate/placebo), and dependent variables Positive Affect, Negative Affect, Calmness, Contentedness, Alertness, and Medical Symptoms, with a follow-up MANOVA of baseline (T1) effects only, with factor Drug and the same dependent variables. Significant effects were further explored with Bonferroni corrected repeated measures ANOVA comparing pre (T1) vs post (T2, T3) intake (Helmert contrast), where alpha = .05/6≈.008. Greenhouse-Geisser correction was applied when the assumption of sphericity was not met. Finally, we assessed whether Listening Span and Impulsivity interacted with the effects of methylphenidate on the self-report rating, by including these covariates in the main MANOVA.

Methylphenidate affected self-report ratings (Time x Drug: *V* = .38, *F*(12,89) = 4.6, *p* < .001), in the absence of baseline (T1) differences between the methylphenidate and placebo groups (T1 only, Drug: *V* = .06, *F*(6,95) = 1.0, *p* = .5). After capsule intake, relative to placebo methylphenidate increased Positive Affect (*F*(1,100) = 19.9, *p* <.001), Alertness (*F*(1,100) = 16.8, *p* <.001), and Medical Symptoms (*F*(1,100) = 9.9, *p* =.002), decreased Calmness (*F*(1,100) = 7.9, *p* =.006), and did not significantly affect Contentedness (*F*(1,100) = 1.4, p=.23) and Negative Affect (*F*(1,100) = 0.4, p=.5). The effects of methylphenidate on the self-report ratings did not significantly interact with Listening Span (Drug x Time x WMspan: *V = 0.1, F(12,87) =0.8, p=.7*) and Impulsivity (Drug x Time x BIS*: V = 0.2, F(12,87) =1.8, p=.063*), which suggests that the methylphenidate-induced changes in mood and medical symptoms were orthogonal to the Listening Span dependent methylphenidate effects we observed on the task of interest here.

## Win-Stay Lose-Shift analyses

For consistency with our previous work, we also assessed trial-by-trial behavioural adjustment following rewards and punishments, reflecting (instantaneous) learning. This is reflected in the tendency to select the same stimulus or instead to shift to a different stimulus, following either a win (win-stay) or a loss (lose-shift). Note that chance level probability to shift is 2/3 and to stay is 1/3, given that there are always 3 stimulus options. Therefore, we computed the proportion of stay after win and after loss, $p(stay|win)$ and $p(stay|loss)$, for which chance level is 1/3. We subtract 1/3 from all scores to allow us to interpret the intercept as a deviation from chance performance. We conducted a three-way repeated-measures ANOVA with factors Outcome (win, loss), Phase (acquisition, reversal) and Drug (methylphenidate, placebo), again with Listening Span total score and Barratt Impulsiveness total score as covariates.

Regarding the degree to which an outcome affected subsequent choice, participants were more likely than chance to repeat a choice on the subsequent trial (Main effect of p(Stay): F(1,99) = 541.3; p<.001, η^2^=.84; (*Main manuscript Figure 1.E*). However, they were less likely to repeat their choice following a Loss than following a Win (Outcome: F(1,99)=302.9, p<.0001, η^2^=.75), indicating subsequent choice was modulated by feedback. This effect was weaker during the reversal phase (Outcome x Phase: F(1,99)=21.6, p<.0001, η^2^=.18), putatively in line with slower learning during reversal. However, there was no significant effect of methylphenidate on either the overall tendency to stay (Drug: F(1,99)=2.2, p=.13, η^2^=.022), nor its modulation by Outcome (Outcome x Drug: F(1,99)=0.4,p=.5, η^2^=.004), nor did this change over time (Outcome x Phase x Drug: F(1,99)=2.4,p=.12, η^2^=.024).

For completeness, there was a trend towards an interaction of Drug x WM on the tendency to stick to a particular choice, regardless of feedback (Drug x WM: F(1,99)=3.04 , p=.084, η^2^=.030). In light of the effect of methylphenidate on inverse learning rate $\phi$as a function of working memory (WM) span, perhaps of interest to note is a near trend effect on feedback sensitivity (i.e. changes in performance driven by feedback on the previous trial), where methylphenidate tended to change the degree of staying following a win versus loss during the acquisition phase (Drug x Outcome x Phase x WM (F(1,99)=2.4, p=.12, η^2^=.023). This effect indexes the degree to which participants follow a win-stay lose-shift strategy, which is effectively a learning rate of 1. This trend effect on win-stay lose-shift may reflect the change in learning rate that is captured by the computational model. However, because a win-stay lose-shift analysis only captures integration of information 1 trial back, this analysis is much less sensitive than our computational approach.

There were no further significant interactions of the effects of methylphenidate predicted by BIS or WM (all p> 0.3).

## Post hoc interaction analyses to characterize main 4-way interaction

Methylphenidate affected performance as a function of WM span (Phase x Drug x WM span: F(1,99)=7.1, p=.009). To understand the nature of this interaction we broke it down into simple effects for each of the factors *Table S2*. We also re-analysed the data excluding RT<200 ms., as these are likely to reflect fast responses where stimulus values were not considered. 51 trials were excluded due to RT’s under 200 ms. We confirm that our conclusions remain unchanged, as reported in *Table S2*.

Table S2. Post hoc simple interaction analyses of WM span mediated effect of methylphenidate.

|  | **All RT** | **RT<200ms excluded** |
| --- | --- | --- |
| **Phase x Drug x WM span** | F(1,99)=7.1, p=.009** | F(1,99)=7.1, p=.009** |
| **Valence x Phase x Drug x WM span** | F(1,99)=5.4, p=0.022* | F(1,99)=5.4, p=0.022* |
| Drug x WM span | |  |
| *Acquisition* | F(1,99)=9.8, p = .002** | F(1,99)=9.8, p =.002** |
| *Reversal* | F(1,99)=0.1, p = .75 | F(1,99)=0.1, p = .75 |
| Phase x Drug x WM span | |  |
| *pReward* | F(1,99)=7.6, p = .007** | F(1,99)=7.6, p = .007** |
| *pAvoidPunish* | F(1,99)=3.7 p = .057 | F(1,99)=3.7, p = .059 |
| *pNeutral ^+^* | F(1,99) =5.4 p = .022* | F(1,99) =5.4 p = .022* |
| Phase x WM span | |  |
| *Methylphenidate* | F(1,99)=4.5, p=.037* | F(1,99)=4.4, p=.038* |
| *Placebo* | F(1,99)=1.3, p=.25 | F(1,99)=1.3, p=.25 |
| ^+^ Note that the neutral stimuli were not included in the original interaction as they are not independent, but are included here for completeness. | | |

## Subject-level optimal parameter estimates for full model space

Table S3. Model evidence and parameter estimates for base model and methylphenidate model families

| **Base model family** | | | | | | |
| --- | --- | --- | --- | --- | --- | --- |
| Model | Param.* | Constraint | Median | Range (25-75%) | pxp_i_ | P(m_i_|data, m) |
| EWA |  |  |  |  | 0.01 | 0.22 |
|  | $\varphi$ | $0\leq\varphi\leq1$ | 0.56 | 0.25 – 0.71 |  |  |
|  | $\rho$ | $0\leq\rho\leq1$ | 0.51 | 0.21 – 0.69 |  |  |
|  | $\beta$ | $0\leq\beta\leq+\infty$ | 3.16 | 2.46 – 4.53 |  |  |
| **EWA+F** |  |  |  |  | **0.91** | **0.40** |
|  | $\varphi$ | $0\leq\varphi\leq1$ | **0.77** | **0.29 – 0.87** |  |  |
|  | $\rho$ | $0\leq\rho\leq1$ | **0.63** | **0.27 – 0.83** |  |  |
|  | $\beta$ | $0\leq\beta\leq+\infty$ | **4.23** | **3.11 – 7.88** |  |  |
|  | $\alpha_{f}$ | ${0\leq\alpha}_{f}\leq1$ | **0.35** | **0.02 – 0.68** |  |  |
| Hybrid |  |  |  |  | 0.0 | 0.10 |
|  | $\kappa$ | $0\leq\kappa\leq1$ | 0.57 | 0.42 – 0.76 |  |  |
|  | $\eta$ | $0\leq\eta\leq1$ | 0.23 | 0.02 – 0.60 |  |  |
|  | $\beta$ | $0\leq\beta\leq+\infty$ | 3.35 | 2.09 – 4.85 |  |  |
| Hybrid+F |  |  |  |  | 0.08 | 0.28 |
|  | $\kappa$ | $0\leq\kappa\leq1$ | 0.35 | 0.18 – 0.69 |  |  |
|  | $\eta$ | $0\leq\eta\leq1$ | 0.22 | 0.05 – 0.71 |  |  |
|  | $\beta$ | $0\leq\beta\leq+\infty$ | 5.73 | 3.06 – 10.03 |  |  |
|  | $\alpha_{f}$ | ${0\leq\alpha}_{f}\leq1$ | 0.29 | 0.02 – 0.68 |  |  |
| **Methylphenidate model family** | | | | | | |
| Model | Param.* | Constraint | Median | Range(25-75%) | pxp_i_ | P(m_i_|data, m) |
| EWA+F+$\boldsymbol{\Delta}_{\boldsymbol{\rho}}$ |  |  |  |  | 0 | 0.13 |
|  | $\varphi$ | $0\leq\varphi\leq1$ | 0.73 | 0.30 – 0.86 |  |  |
|  | $\rho_{Placebo}$ | $0\leq\rho_{Placebo}\leq1$ | 0.56 | 0.23 – 0.82 |  |  |
|  | $\rho_{MPH}$ | $0\leq\rho_{MPH}\leq1$ | 0.52 | 0.23 – 0.78 |  |  |
|  | $\beta$ | $0\leq\beta\leq+\infty$ | 4.79 | 3.34– 7.83 |  |  |
|  | $\alpha_{f}$ | ${0\leq\alpha}_{f}\leq1$ | 0.28 | 0.02 – 0.65 |  |  |
| **EWA+F+**$\boldsymbol{\Delta}_{\boldsymbol{\varphi}}$ |  |  |  |  | **0.98** | **0.42** |
|  | $\varphi_{Placebo}$ | $0\leq\varphi_{Placebo}\leq1$ | **0.70** | **0.26 – 0.86** |  |  |
|  | $\varphi_{MPH}$ | $0\leq\varphi_{MPH}\leq1$ | **0.70** | **0.33 – 0.84** |  |  |
|  | $\rho$ | $0\leq\rho\leq1$ | **0.56** | **0.21 – 0.79** |  |  |
|  | $\beta$ | $0\leq\beta\leq+\infty$ | **4.46** | **3.13 – 7.94** |  |  |
|  | $\alpha_{f}$ | ${0\leq\alpha}_{f}\leq1$ | **0.22** | **0.01 – 0.62** |  |  |
| EWA+F+$\Delta_{\alpha_{f}}$ |  |  |  |  | 0 | 0.12 |
|  | $\varphi$ | $0\leq\varphi\leq1$ | 0.66 | 0.31 – 0.86 |  |  |
|  | $\rho$ | $0\leq\rho\leq1$ | 0.61 | 0.25 – 0.82 |  |  |
|  | $\beta$ | $0\leq\beta\leq+\infty$ | 4.80 | 3.18 – 7.43 |  |  |
|  | ${\alpha_{f}}_{Placebo}$ | $0\leq{\alpha_{f}}_{Placebo}\leq1$ | 0.26 | 0.02 – 0.60 |  |  |
|  | ${\alpha_{f}}_{MPH}$ | $0\leq{\alpha_{f}}_{MPH}\leq1$ | 0.21 | 0.02 – 0.49 |  |  |
| EWA+F+$\Delta_{\boldsymbol{\beta}}$ |  |  |  |  | 0 | 0.08 |
|  | $\varphi$ | $0\leq\varphi\leq1$ | 0.68 | 0.28 – 0.85 |  |  |
|  | $\rho$ | $0\leq\rho\leq1$ | 0.55 | 0.20 – 0.83 |  |  |
|  | $\beta_{Placebo}$ | $0\leq\beta_{Placebo}\leq1$ | 4.21 | 2.76 – 8.54 |  |  |
|  | $\beta_{MPH}$ | $0\leq\beta_{MPH}\leq1$ | 4.32 | 2.90 – 9.37 |  |  |
|  | $\alpha_{f}$ | ${0\leq\alpha}_{f}\leq1$ | 0.25 | 0.02 – 0.64 |  |  |
| ^*^ A weakly informative Gaussian prior was used for all parameters ($x\sim N(\mu,\sigma^{2})$ where the mean value $\mu=0$ and the variance $\sigma^{2}=10$). According to theoretical constraints of parameters, sigmoid or exponential transformations are applied. | | | | | | |

## Model space validation

Here, we verify that the conclusion that methylphenidate only affects the inverse learning rate $\varphi$ did not resulted from our assumption that methylphenidate was allowed to affect only a single parameter in the model, thereby ‘forcing’ the effect of methylphenidate to load on a single parameter. First, we fitted a model where all parameters were estimated separately for the methylphenidate and placebo sessions, $Model\_\Delta_{All}$. For each parameter, we then examined whether this parameter was a) significantly different under methylphenidate and placebo sessions, and b) whether their difference was predicted by working memory span. There were no significant differences between placebo and methylphenidate parameter estimates for any of the parameters (*Table S4*). In addition to replicating the WM correlation with $\Delta_{\varphi}$, there was also a weaker correlation with $\Delta_{\rho}$. We therefore formulated a second, more reduced, model, where we allowed only $\rho$ and $\varphi$ to vary. We then included both new models in the full model comparison, which showed that our original model proved a far better explanation of the data than either of these new models (*Figure S1A*, PEP = 0.83). Furthermore, in the model that allowed both $\rho$ and $\varphi$ to vary, $\Delta_{\rho}$ did not longer correlate with WM span, while $\Delta_{\varphi}$ did. Taken together, this expansion of model space confirms that only the inverse learning rate $\varphi$is affected by methylphenidate, and that this effect of interest is present across different formulations of the model. Finally, we assessed whether the estimates of $\varphi$ were strongly shaped by the presence or absence of other methylphenidate dependent parameters, by correlating the difference in $\varphi$ estimates between methylphenidate and PLA conditions, across the different models. Reassuringly these were highly correlated (*Figure S1B*).

In all, these results emphasise that the model with one parameter, $\Delta_{\varphi}$, is sufficient to capture the effect of methylphenidate on the reversal learning task, as presented in the main text.

Table S4. Analysis of parameter differences between methylphenidate and placebo: Paired t-test for the main effect (ME) and correlation with working memory span (WM).

| **Model** | **Parameter** | **ME p-value** | **WM p-value** | **WM R (correlation coeff.)** |
| --- | --- | --- | --- | --- |
| $Model\_\Delta_{\varphi}$ | $\varphi$ | .7 | .043* | 0.2 |
| $Model\_\Delta_{All}$ | $\varphi$ | 1 | .004** | 0.28 |
|  | $\rho$ | .8 | .02* | 0.22 |
|  | $\alpha_{f}$ | .7 | .4 | 0.09 |
|  | $\beta$ | .5 | .9 | 0 |
| $Model\_\Delta_{\rho,\varphi}$ | $\varphi$ | .6 | .02* | 0.22 |
|  | $\rho$ | .2 | .7 | -0.03 |


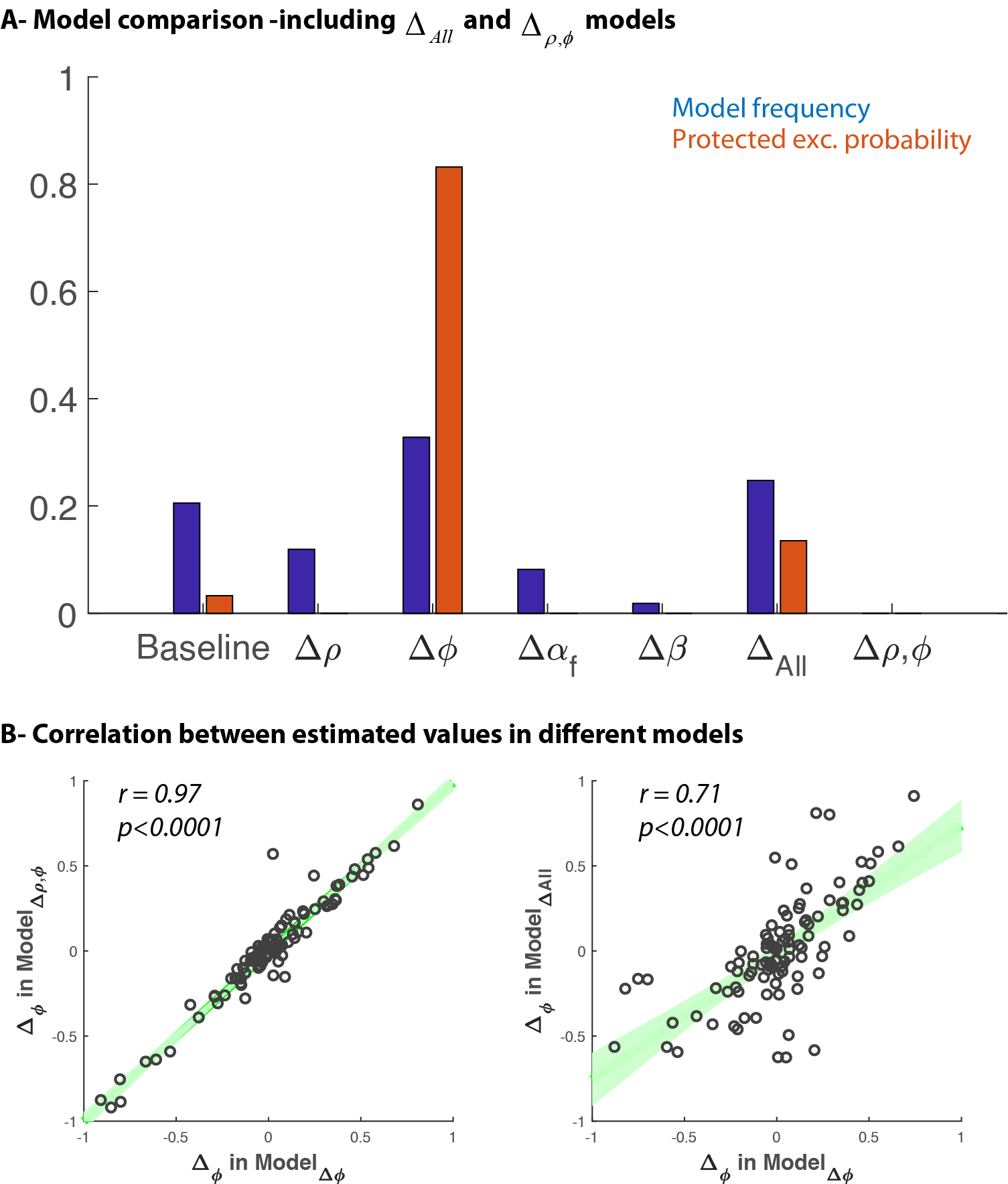


**Figure S1. A. Model comparison including the 2 new models into the ‘old’ model space.** The original model where only $\varphi$ is affected by methylphenidate wins convincingly (PEP=0.83). **B. Correlation between estimated values in different models.** Estimated values for $\Delta_{\varphi}$ in ${Model\_\Delta}_{\varphi}$ are very close to the estimated values in ${Model\_\Delta}_{\rho,\varphi}$ (r=0.97; p<.0001)**.** Also, Estimated values for $\Delta_{\varphi}$ in ${Model\_\Delta}_{\varphi}$ are highly correlated with the estimated values in ${Model\_\Delta}_{All}$ (r=0.71; p<.0001).

## Performance comparison for 2 vs 3 choice option PRL task

At first sight, a puzzling aspect of our findings was that methylphenidate affected performance during acquisition, rather than reversal, in contrast to previous findings where dopamine affected perseverative behaviour (cf. (den Ouden et al. 2013)). Instead, we observed an effect of methylphenidate on initial acquisition of the reward contingencies. We hypothesise that through introducing and additional ‘neutral’ choice option (with 50/50 reward/punishment contingencies), we have likely changed the dynamics and difficulty of the task. Specifically, the crucial distinction for optimal performance changed from dissociating a 70/30 from a 30/70 cue, while in the 3-choice task, this required dissociating a 75/25 from a 50/50 option. We explore the effects of this change below.

In order to assess the impact of adding a neutral choice option on the reversal performance, we compared average behaviour of participants for 2-option version of the task ((den Ouden et al. 2013), N=204) and the current, 3-option, task. Supplemental *Figure S2A* displays average trial-by-trial choice probability for the mostly rewarded stimulus during the acquisition phase, which becomes the mostly punished stimulus during the reversal phase. For the 2-choice task, the ratio of reward/punishment for the Rewarded choice is 70:30 and in the current version it is 75:25. In both versions, participants learned to make the correct selection for the acquisition phase. However, reversal performance was much more rapid during the 3-choice task.

When fitting and comparing parameter estimates to both versions of the task for the winning Experience-Weighted Attraction (EWA) model (described in the main text), there was a striking difference in (inverse) learning rate parameter $\varphi$, which was much higher in the current 3-choice task. This meant that the effective learning rate was much lower, i.e. people integrated information over a longer effective time window. In contrast, the winning experience weight parameter ($\rho$) and decision noise ($\beta$) estimates have very similar values (supplemental *Table S5*). This holds both when fitting the EWA model that was optimal in the 2-choice PRL task, but also when fitting the winning model for the 3-choice PRL task that included a forgetting factor ($\alpha_{f}$).

Table S5. Median (25%-75% Range) parameter estimates for the 2- versus 3-choice PRL task. The key difference between these tasks is that the inverse learning rate parameter $\varphi$ is much higher (i.e. slower learning) for the 3-option model, while other parameter values were relatively unchanged. This difference illustrates that subjects adjust their learning rates to the task, as the optimal learning rate in the 3 option task is indeed lower.

| **Model** | $\varphi$ | $\rho$ | $\beta$ |
| --- | --- | --- | --- |
| 2 option – EWA | 0.33 (0.10 – 0.62) | 0.62 (0.28 – 0.88) | 4.69 (2.62 – 7.35) |
| 3 option – EWA | 0.56 (0.25 – 0.71) | 0.51 (0.21 – 0.69) | 3.16 (2.46 – 4.53) |
| 3 option – EWA+F | 0.77 (0.29 – 0.87) | 0.63 (0.27 – 0.83) | 4.23 (3.11 – 7.88) |


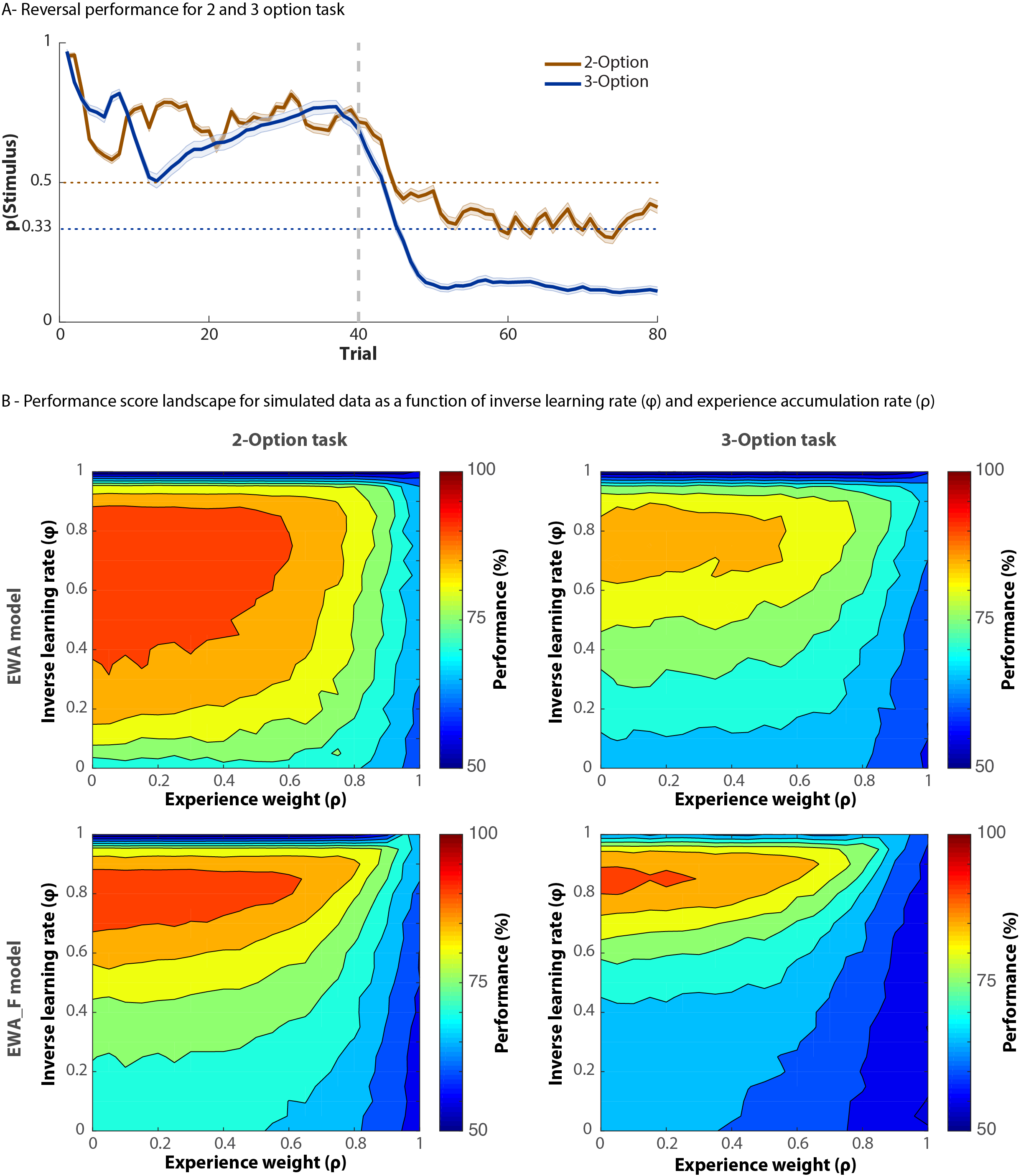


**Figure S2. A. Reversal performance for 2 and 3 option task.** Trial-by-trial averaged probability of selecting the stimulus R-P$S_{1}$, which was mostly rewarded during the acquisition phase and punished during the reversal phase. For illustration, we used a sliding window with 5-trial width. Participants learned to make the correct selection for the acquisition phases in both task, but showed relatively rapid reversal during the 3-option task. The dashed lines indicate chance level choice for each task. Note that the 2-option task had a fixed feedback sequence while the 3-option version did not, resulting in smoother curves when averaging over participants. **B.** **Performance score landscape for simulated data as a function of inverse learning rate ($\boldsymbol{\varphi}$) and experience accumulation rate ($\boldsymbol{\rho}$).** The key observation is that the range of optimal $\varphi$ is much wider for the 2-option relative to the 3-option model, where for the latter, performance steeply drops off as soon as $\varphi$ decreases. Also, note also that ‘forgetting’ of the unchosen option helps performance in both task versions. Importantly, the pattern of performance score sensitivity as a function of $\varphi$ is constant across the models (with and without forgetting).

### Optimal learning rate reduced in 3-choice PRL task

In order to quantify the intuition that whether this change in learning rate was adaptive in the novel task, we computed the optimal learning rate for each task version, for both winning models (EWA vs. EWA with forgetting). In order to find the optimal values for $\varphi$ and $\rho$ we simulated performance score for the entire [0 1] range of these two parameters with step size = 0.05. For all other parameters, we used the median value of the fitted parameters across participants (EWA: $\beta=4.5$ and EWA+F: $\beta=4.5$ and $\alpha_{f}=0.35$). We ran 100 simulations for each parameter set. We computed a (normalized) performance score based on the likelihood of receiving positive feedback for each of the stimulus options:

2-choice PRL task

| $Performance=\frac{(0.70\times N_{Rewarded}+0.30\times N_{Punished}-0.30\times N_{Trials})}{0.70\times N_{Trials}-0.30\times N_{Trials}}\times100$ | (5) |
| --- | --- |

3-choice PRL task

| $Performance=\frac{(0.75\times N_{Rewarded}+0.50\times N_{Neutral}+0.25\times N_{Punished}-0.25\times N_{Trials})}{0.75\times N_{Trials}-0.25\times N_{Trials}}\times100$ | |
| --- | --- |
|  | (6) |

Where $[N_{Rewarded},N_{Neutral},N_{Punished}]$ $[N_{\mathrm{Rewarded}} N_{\mathrm{Neutral}} N_{\mathrm{Punished}}]$is the number of trials which each stimulus is selected and $N_{Trials}$ is the total number of trials (80 for both tasks). The performance score normalized to a minimum of 0 when the punished stimulus was chosen on all trials, and a maximum of 100 when the rewarded stimulus was chosen on all trials.

In line with the observed participant behaviour across the two tasks, subjects show a lower effective learning rate (high $\varphi$) for the 3-option than the 2-option task. Moreover, a higher learning rate (low $\varphi$) was associated with lower performance scores (*Figure S1B*). In contrast, for the 2-choice model the range of learning rates that result in optimal performance is much wider. This optimal lower learning rate in the 3-option task can be understood when realising that optimal performance on the 3-choice tasks requires participants to dissociate between a 50/50 and a 75/25 reward/punishment cue. In contrast, in the 2-choice task the key dissociation was between a 70/30 and 30/70 cue. For the latter, integration over fewer choice-outcome observations, i.e. a narrower window to the past, so a higher learning rate, is required to distinguish the optimal action. Of note, this reduction in learning rate is also reflected in the raw behavioural scores, with a much lower tendency to shift following a loss. Indeed, lose-shift behaviour is below chance level, meaning that even after a loss, people are more likely than chance to repeat their previous choice (cf. *Figure 1*, main text), compared to above-chance lose-shift rates in the 2-option task (c.f. *Figure 2B;* (den Ouden et al. 2013)).

# References

Barratt W (2006) The Barratt Simplified Measure of Social Status (BSMSS): Measuring SES. Unpubl manuscript, Indiana State Univ

Beck AT, Steer RA, Ball R, Ranieri WF (1996) Comparison of Beck Depression Inventories-IA and-II in psychiatric outpatients. J Pers Assess 67:588–597. https://doi.org/10.1207/s15327752jpa6703_13

Behrens TEJ, Woolrich MW, Walton ME, Rushworth MFS (2007) Learning the value of information in an uncertain world. Nat Neurosci 10:1214–1221. https://doi.org/10.1038/nn1954

Bond A, Lader M (1974) The use of analogue scales in rating subjective feelings. Br J Med Psychol 47:211–218. https://doi.org/10.1111/j.2044-8341.1974.tb02285.x

Buckholtz JW, Treadway MT, Cowan RL, et al (2010) Dopaminergic network differences in human impulsivity. Science (80- ) 329:532. https://doi.org/10.1126/science.1185778

Cacioppo JT, Petty RE, Kao CF (1984) The efficient assessment of need for cognition. J Pers Assess 48:306–307

Camerer C, Ho TH (1999) Experience‐weighted attraction learning in normal form games. Econometrica 67:827–874

Canty-Mitchell J, Zimet GD (2000) Psychometric properties of the Multidimensional Scale of Perceived Social Support in urban adolescents. Am J Community Psychol 28:391–400. https://doi.org/10.1023/A:1005109522457

Carver CS, White TL (1994) Behavioral inhibition, behavioral activation, and affective responses to impending reward and punishment: The BIS/BAS Scales. J Pers Soc Psychol 67:319–333. https://doi.org/10.1037//0022-3514.67.2.319

Clatworthy PL, Lewis SJG, Brichard L, et al (2009) Dopamine release in dissociable striatal subregions predicts the different effects of oral methylphenidate on reversal learning and spatial working memory. J Neurosci 29:4690–6. https://doi.org/10.1523/JNEUROSCI.3266-08.2009

Cook JL, Den Ouden HEM, Heyes CM, Cools R (2014) The social dominance paradox. Curr Biol 24:2812–2816. https://doi.org/10.1016/j.cub.2014.10.014

Cools R, D’Esposito M (2011) Inverted-U-shaped dopamine actions on human working memory and cognitive control. Biol Psychiatry 69:e113–e125. https://doi.org/10.1016/j.biopsych.2011.03.028

Cools R, Gibbs SE, Miyakawa A, et al (2008) Working memory capacity predicts dopamine synthesis capacity in the human striatum. J Neurosci 28:1208–12. https://doi.org/10.1523/JNEUROSCI.4475-07.2008

Daneman M, Carpenter PA (1980) Individual differences in working memory and reading. J Verbal Learning Verbal Behav 19:450–466. https://doi.org/10.1016/s0022-5371(80)90312-6

Daw ND (2011) Trial-by-trial data analysis using computational models. In: Delgado M, Phelp E, Robbins T (eds) Decision Making, Affect, and Learning. Oxford University Press, pp 3–38

De Weerd S, Van der Bij AK, Braspenning JCC, et al (2001) Psychological impact of preconception counseling: Assessment of anxiety before and during pregnancy. Community Genet 4:129–133

Dempster AP, Laird NM, Rubin DB (1977) Maximum likelihood from incomplete data via the EM algorithm. J R Stat Soc Ser B 39:1–22. https://doi.org/10.1111/j.2517-6161.1977.tb01600.x

den Ouden HEM, Daw ND, Fernandez G, et al (2013) Dissociable effects of dopamine and serotonin on reversal learning. Neuron 80:1090–1100. https://doi.org/10.1016/j.neuron.2013.08.030

Frank MJ, Claus ED (2006) Anatomy of a decision: Striato-orbitofrontal interactions in reinforcement learning, decision making, and reversal. Psychol Rev 113:300–326. https://doi.org/10.1037/0033-295X.113.2.300

Franken IHA, Muris P, Rassin E (2005) Psychometric properties of the Dutch BIS/BAS scales. J Psychopathol Behav Assess 27:25–30. https://doi.org/10.1007/s10862-005-3262-2

Ito M, Doya K (2009) Validation of decision-making models and analysis of decision variables in the rat basal ganglia. J Neurosci 29:9861–9874. https://doi.org/10.1523/JNEUROSCI.6157-08.2009

Jordan MI, Ghahramani Z, Jaakkola TS, Saul LK (1998) An introduction to variational methods for graphical models. Mach Learn 37:183–233. https://doi.org/10.1023/A:1007665907178

Kalma AP, Visser L, Peeters A (1993) Sociable and aggressive dominance: Personality differences in leadership style? Leadersh Q 4:45–64. https://doi.org/10.1016/1048-9843(93)90003-C

Kass, R E, Raftery, A E (1995) Bayes factors. J Am Stat Assoc 90:773–795

Kim JH, Son YD, Kim HK, et al (2014) Dopamine D 2/3 receptor availability and human cognitive impulsivity: a high-resolution positron emission tomography imaging study with [11 C]raclopride. Acta Neuropsychiatr 26:35–42. https://doi.org/10.1017/neu.2013.29

Kimberg DY, D’Esposito M (2003) Cognitive effects of the dopamine receptor agonist pergolide. Neuropsychologia 41:1020–1027. https://doi.org/10.1016/S0028-3932(02)00317-2

Kimberg DY, D’Esposito M, Farah MJ (1997) Effects of bromocriptine on human subjects depend on working memory capacity. Neuroreport 8:3581–3585. https://doi.org/10.1097/00001756-199711100-00032

Landau SM, Lal R, O’Neil JP, et al (2009) Striatal dopamine and working memory. Cereb Cortex 19:445–454. https://doi.org/10.1093/cercor/bhn095

Lee B, London ED, Poldrack RA, et al (2009) Striatal dopamine D2/D3 receptor availability is reduced in methamphetamine dependence and is linked to impulsivity. J Neurosci 29:14734–14740. https://doi.org/10.1523/JNEUROSCI.3765-09.2009

Li J, Schiller D, Schoenbaum G, et al (2011) Differential roles of human striatum and amygdala in associative learning. Nat Neurosci 14:1250–1252. https://doi.org/10.1038/nn.2904

Patton JH, Stanford MS, Barratt ES (1995) Factor structure of the Barratt impulsiveness scale. J Clin Psychol 51:768–774. https://doi.org/10.1002/1097-4679(199511)51:6<768

Piray P, Dezfouli A, Heskes T, et al (2019a) Hierarchical Bayesian inference for concurrent model fitting and comparison for group studies. PLOS Comput Biol 15:e1007043. https://doi.org/10.1371/journal.pcbi.1007043

Piray P, Ly V, Roelofs K, et al (2019b) Emotionally aversive cues suppress neural systems underlying optimal learning in socially anxious individuals. J Neurosci 39:1445–1456. https://doi.org/10.1523/JNEUROSCI.1394-18.2018

Reeves SJ, Polling C, Stokes PRA, et al (2012) Limbic striatal dopamine D2/3 receptor availability is associated with non-planning impulsivity in healthy adults after exclusion of potential dissimulators. Psychiatry Res - Neuroimaging 202:60–64. https://doi.org/10.1016/j.pscychresns.2011.09.011

Rigoux L, Stephan KE, Friston KJ, Daunizeau J (2014) Bayesian model selection for group studies - Revisited. Neuroimage 84:971–985. https://doi.org/10.1016/j.neuroimage.2013.08.065

Salthouse TA, Babcock RL (1991) Decomposing adult age differences in working memory. Dev Psychol 27:763–776. https://doi.org/10.1037/0012-1649.27.5.763

Schulte-Van Maaren YWM, Carlier IVE, Zitman FG, et al (2013) Reference values for major depression questionnaires: The Leiden Routine Outcome Monitoring Study. J Affect Disord 149:342–349. https://doi.org/10.1016/j.jad.2013.02.009

Spielberger CD, Gorsuch R, Lushene P, et al (1983) Manual for the State-Trait Anxiety Inventory. Consulting Psycholgists Press, Palo Alto, CA

Stephan KE, Penny WD, Daunizeau J, et al (2009) Bayesian model selection for group studies. Neuroimage 46:1004–1017. https://doi.org/10.1016/j.neuroimage.2013.08.065

van der Schaaf ME, van Schouwenburg MR, Geurts DEM, et al (2014) Establishing the dopamine dependency of human striatal signals during reward and punishment reversal learning. Cereb Cortex 24:633–642. https://doi.org/10.1093/cercor/bhs344

Watson D, Clark LA, Tellegen A (1988) Development and validation of brief measures of positive and negative affect: The PANAS scales. J Pers Soc Psychol 54:1063–1070. https://doi.org/10.1037/0022-3514.54.6.1063

Westbrook A, Kester D, Braver TS (2013) What is the subjective cost of cognitive effort? Load, trait, and aging effects revealed by economic preference. PLoS One 8:e68210. https://doi.org/10.1371/journal.pone.0068210

Zimet GD, Dahlem NW, Zimet SG, Farley GK (1988) The multidimensional scale of perceived social support. J Pers Assess 52:30–41. https://doi.org/10.1207/s15327752jpa5201_2
